# Multidimensional attributes expose Heider balance dynamics to measurements

**DOI:** 10.1038/s41598-023-42390-w

**Published:** 2023-09-20

**Authors:** Joanna Linczuk, Piotr J. Górski, Boleslaw K. Szymanski, Janusz A. Hołyst

**Affiliations:** 1 Faculty of Physics, Warsaw University of Technology, Koszykowa 75, 00-662 Warsaw, Poland; 2 NEST Center, Dept. Computer Science, Rensselaer Polytechnic Institute, 110 8th Street, Troy, NY 12180-3590 USA; 3 Academy of Social Sciences, Henryka Sienkiewicza 9, 90-113 Łódź, Poland

**Keywords:** Complex networks, Computational science

## Abstract

Most of studied social interactions arise from dyadic relations. An exception is Heider Balance Theory that postulates the existence of triad dynamics, which however has been elusive to observe. Here, we discover a sufficient condition for the Heider dynamics observability: assigning the edge signs according to multiple opinions of connected agents. Using longitudinal records of university student mutual contacts and opinions, we create a coevolving network on which we introduce models of student interactions. These models account for: multiple topics of individual student opinions, influence of such opinions on dyadic relations, and influence of triadic relations on opinions. We show that the triadic influence is empirically measurable for static and dynamic observables when signs of edges are defined by multidimensional differences between opinions on all topics. Yet, when these signs are defined by a difference between opinions on each topic separately, the triadic interactions’ influence is indistinguishable from noise.

## Introduction

Formation and evolution of human relations are complex processes influenced by a multitude of factors^1–14^. Most agent-based models describing dynamics of social structures focus on dyadic interactions. However, in many systems higher-order relations are also important^15–18^. Heider Balance Theory (HBT, also known as Structural Balance Theory) was proposed in^1,19^. It postulates that people building social networks follow the well-known rules: *a friend of my friend* or *an enemy of my enemy is my friend*, but *a friend of my enemy* or *an enemy of my friend is my enemy*. This creates relations within fully-connected subgraphs of a social network, where a dyadic relationship of one person to another defines this person’s relations to friends and enemies of another person. Edges connecting a person to friends are assigned a positive sign, while those connecting to enemies get a negative sign. This allows us to recognize balanced and unbalanced higher-order fully-connected relationship subgraphs. The balanced subgraphs have all nodes in relationships compliant with the HBT rules, thus, they are considered more stable than the unbalanced ones, each containing at least one pair of nodes breaking the HBT rules. Over the years, HBT has become a well-established theory. It has been studied primarily in the context of network science among triads—fully-connected groups of three agents whose social connections are either positive or negative^20–28^. For triads, the determination of balance is simple—a triad with the positive product of signs of its edges is balanced, while the negative product indicates that it is unbalanced.

Nowadays, broad data availability allows experiments to assess postulates of HBT in different environments, ranging from little communities to large scale social networks^15,16,29–34^. The often-observed discrepancy between HBT and data is hypothesized^29,35–37^ to result from other competing processes driving the social network evolution.

In HBT, a dyadic relationship of friendship or enmity can be derived from the attributes of two nodes involved in it^38,39^. A popular form of such derivation are social systems of agents having opinions in which friendship is assigned to an edge joining two agents with similar opinions. Here, we consider HBT edges of this type. Thus, interactions, including triadic ones, form under the influence of an agreement or disagreement on individual attributes, e.g., personal opinions on important topics. On the other hand, human interactions influence people’s beliefs and interests^40^. Therefore, both processes coevolve and influence each other^10,12,41–43^. Coevolution of HBT and agents’ attributes allows analysis of system polarization or consensus^28,43–51^. Usually, one-dimensional (scalar) variables define states of nodes and links. A sign of the edge directly depends on the states of its endpoints and individual or multidimensional distances between the endpoints’ opinions^28,45^. In^46^, the authors show that multiple attributes are important in studies of a convergence of a polarized group of agents to an in-group consensus.

Our manner of deriving signs of edges using similarities between agents resembles the consequences of homophily theory, which assumes that similar people like each other and tend to be connected. Homophily is often studied in unsigned networks and the density of connections between similar agents is compared to the density between different ones. There is no doubt homophily is an important process governing relations in social networks^35^, however, there are varying results on how homophily by itself sufficiently explains social phenomena. It has been shown that the observed level of homophily is amplified by triadic closure^11^. On the other hand, similarities and differences between opinions are not enough to obtain collective behavior of polarization^14^. For some data sets, dyadic interactions based on homophily reproduce patterns expected to result from HBT^52^. In^18^, an introduced higher-order measure allows a more thorough analysis of social group dynamics as compared to standard homophily methods.

Here, we study triadic interactions postulated by HBT in the case of a system defined by a social data set gathered over a two-year span among undergraduates at a university^53^. We check the mutual influence of students’ opinions on specific topics on their social interactions defined by mobile communications. The main goal of this paper is to find the conditions under which the relations between nodes postulated by Heider Balance Theory will be exposed for investigation. We show that static and dynamical behavior of this system in terms of changes in the numbers of balanced and unbalanced triads can be explained only when all components of a set of singular opinions are considered together. When considered separately, these attributes do not explain observed students’ communication patterns. It means that relationships postulated by Heider Balance Theory are measurable when multidimensional attributes are involved but are insignificant when only single opinions are considered. We present the results of using three different statistical approaches, which model the evolution of students’ opinions as individual singular or collective multidimensional opinions. We also introduce an agent-based model with triad dynamics that can reproduce triad transition statistics better than a model of randomized processes. The results demonstrate that multidimensional opinions are sufficient to observe the interactions postulated by HBT in the studied social network of university students.

## Results

### Data set and constructed sign networks

The data used in the research were obtained during the NetSense experiment^53,54^ conducted at the University of Notre Dame. The experiment tracked 204 freshmen who had joined the University in the fall of 2011. Each student received a smartphone for use during the experiment, which lasted six terms. The students took questionnaires every term answering, among others, eight questions about their opinions on: abortion, death penalty, euthanasia, gay marriage, homosexuality, marijuana use, politics, and premarital sex. From the initial group of students who signed up for the experiment, only 108 completed all six surveys. We used their data to obtain the results presented here.

We perform the same steps in the data preparation process as in^54^. The gathered data is divided into two parts. The first part contains students’ personal attributes and opinions on important social topics collected in questionnaires. In general, for each topic, students could declare their opinion about it by selecting one of the values coded as: $-1$ (being against), 0 (unsure), or $+1$ (in support), see Supplementary Materials (SM), Table S1. The second part of the data set contains records of call and message events used to create an evolving student communication network. The network consists of six snapshots, one for each term. Nodes represent students and links represent interactions between nodes by calls or messages. In each term, an edge joins every pair of students who called or messaged each other at least once during the term. Summing up, combining information about changes of students’ opinions and their contacts, we created a network in which both the structure and node states evolve from term to term.

**Figure 1:**
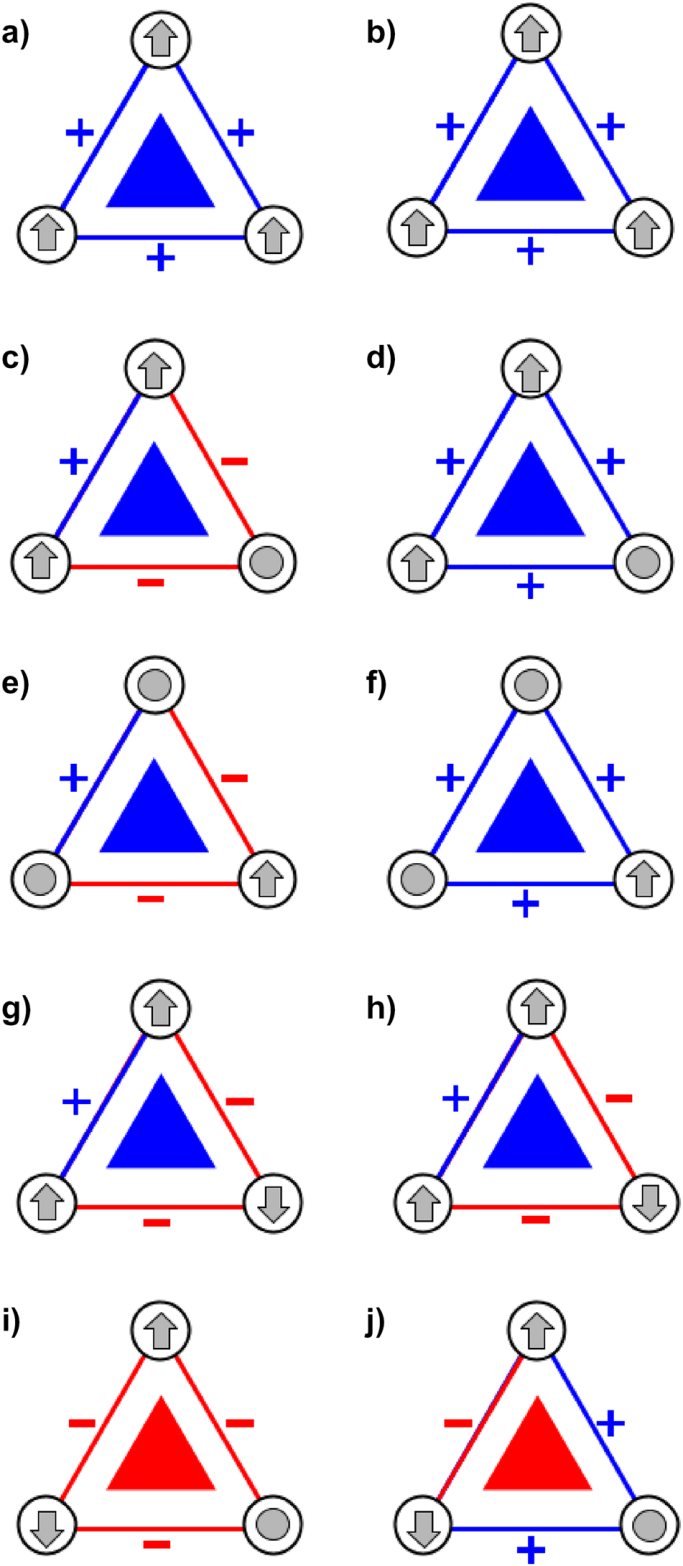
Different ways of defining signs in simple triads. Agents’ opinions are depicted as symbols: $\varvec{\uparrow }$ (*in support*),  (*unsure*), $\varvec{\downarrow }$ (*against*). Edge color and symbol next to it inform about the sign of the edge (blue and  is for positive; red and  for negative). The color of the filled triangle corresponds to the triad’s balance type – blue is for balanced and red for unbalanced. The left column corresponds to the rule that an edge is positive only when it connects endpoints with the same opinion. The right column corresponds to the rule that an edge is a negative only when it connects endpoints holding two different *extremist* opinions. Both these interpretations lead to the same type of triad’s balance. A triad is balanced when at least two of its nodes hold the same opinion (triads a and b, and c to h) . An unbalanced triad arises only when all its three agents hold different opinions (triads i and j).

**Figure 2:**
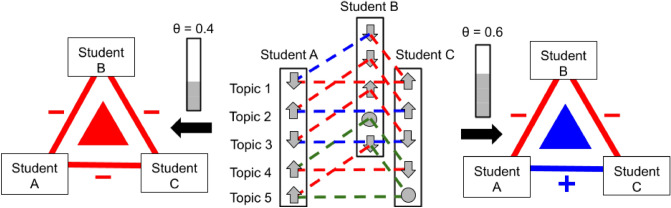
Multidimensional triad construction. Heider Balance Theory focuses on triads—fully connected groups of three agents. We consider triads separately in each study term. If three students A, B and C communicate with each other in the considered term, they create a triad. Each of the students holds one of three opinions: *in support, against* (depicted as upwards or downwards arrows), *unsure* (depicted as a circle) on a few topics—components of multidimensional opinion vectors (the figure lists five topics). For each topic, the difference between two opposite *extremist* opinions is $\pm 2$ (red line), between *extremist* and *centrist* is $\pm 1$ (green line), and it is 0 (blue line) for the same opinion. The normalized to range [0, 1] sum of absolute differences between opinions over all topics is a social distance between students. If the distance is no larger than the assumed tolerance $\Theta$, the edge sign is positive. Cases for different values of tolerance $\Theta$ are depicted on the side panels. Left panel presents the case for the tolerance $\Theta =0.4$. The tolerance is small and all edges in the multidimensional triad are negative, resulting in an unbalanced triad (depicted in red). The right panel corresponds to $\Theta =0.6$. With such tolerance, students A and C are similar enough—distance between them is smaller than the tolerance—so the edge is positive. In this case, the multidimensional triad is balanced (depicted in blue).

Going beyond the processing described in^54^, we compute signs defined by the differences between opinions of connected students and assign them to the corresponding edges. Signs $+1$ or $-1$ signify positive or negative relations. We focus on triads, which are three-node fully-connected subgraphs. We analyze two types of triads: simple, whose state depends on a difference of opinions for an individual topic (cf. Fig. 1) and multidimensional triads, whose state depends on multidimensional differences between opinions about the entire set of topics, see Fig. 2. Sign definitions are fully described in the “Methods” Section.

Signs in multidimensional triads are governed by the parameter $\Theta$, called tolerance. The tolerance $\Theta$ is a model parameter, and we shall explore our data checking how the variation of this tolerance impacts observed patterns of social structures. This parameter defines the level of opinion distance at which a link is assigned a negative instead of positive polarization. This means that a low value of $\Theta$ indicates high sensitivity of the measure to differences meaning that with a small difference of opinions an edge is likely to be labeled as a negative one. While having a large value of $\Theta$, only agents with very different opinions will be classified as different and thus links are usually classified as positive. Having more positive links in a randomly generated network, one obtains more balanced triads. Therefore, one can expect that with increasing $\Theta$, the count of balanced triads will also be growing. We shall show that there exists an optimal region of model parameters where Heider interactions are visible.

### Static properties of triads

**Figure 3:**
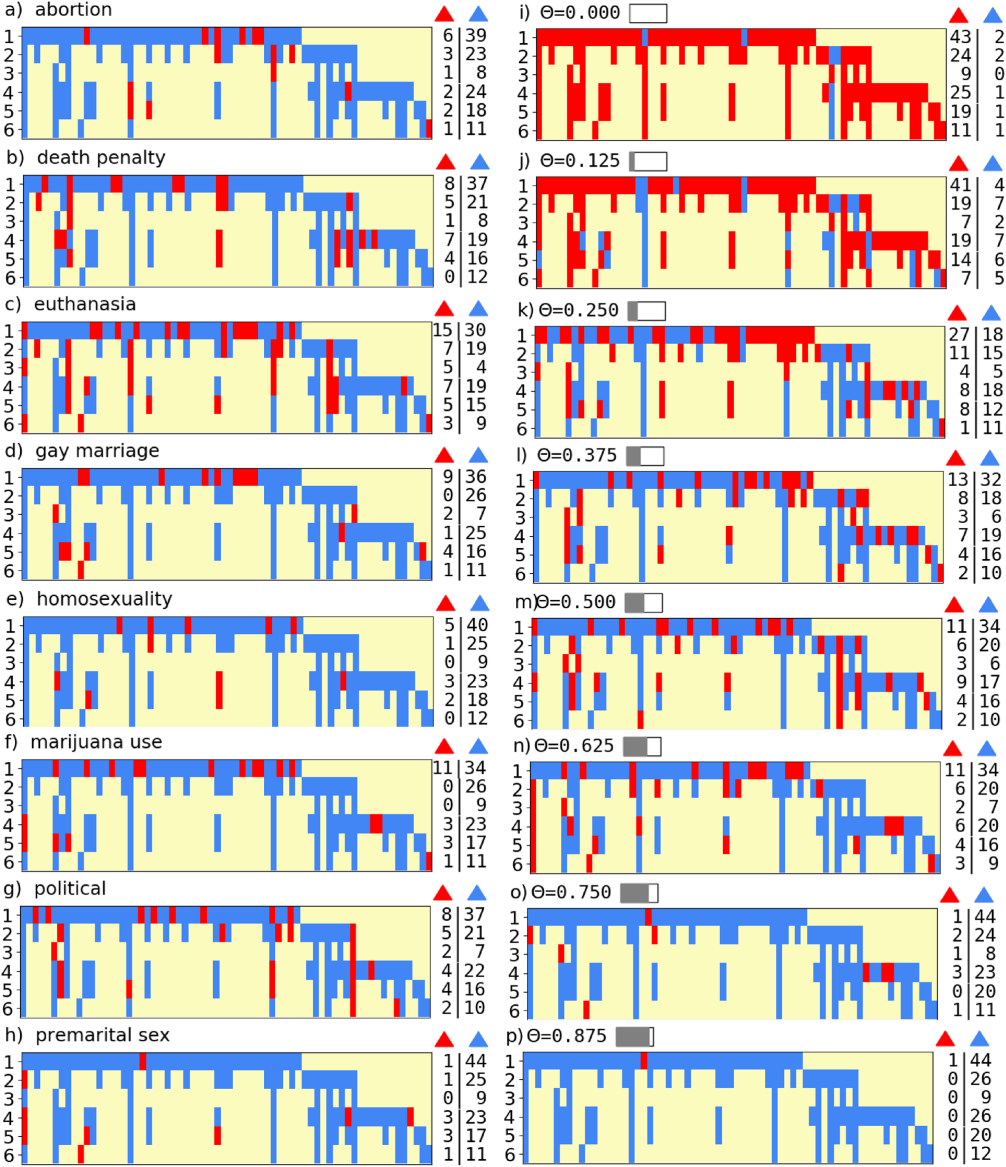
Persistence of triads over terms. Subfigures capture persistence and change in students’ triad status throughout six terms. Each subfigure corresponds to the evolving network with differently defined signs. Left panels (**a**–**h**) show the persistence of all existing simple triads for different topics. Most triads are balanced yet persist only for a few terms. Right panels (**i**–**p**) illustrate the persistence of multidimensional triads for different tolerances. For each subfigure, the six study terms are marked on the y-axis and each column represents a distinct triad (all triads existing in available data are presented). Blue and red stripes indicate the status of triads of each type: balanced and unbalanced, respectively, while yellow ones mean that a triad did not exist in that term. The right margins of the subfigures show the number of triads of each type in each term.

After enumerating all existing simple triads corresponding to different attributes in our data set, we present them in Fig. 3a–h. For all topics, most simple triads are balanced. Yet, it is not clear which topics are crucial for students’ connection evolution. This difficulty motivated us to study multidimensional triads, which consider all topics and the value of tolerance. For each term, we tabulated the number of multidimensional triads in each state (see Fig. 3i–p).

**Figure 4:**
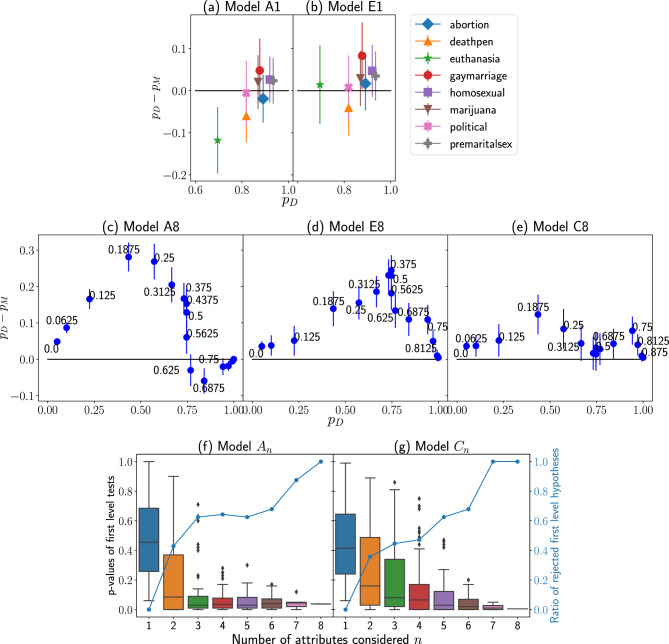
Densities of balanced triads found in NetSense data are not explained by random models when multidimensional opinions are assumed. The plots (a-e) show the comparison of empirical probability $p_D$ of balanced triads obtained from NetSense data and corresponding empirical probabilities ($p_M = \{p_{A_1},p_{E_1},p_{A_8},p_{E_8},p_{C_8}\}$) based on our statistical models that do not consider Heider Balance influence. We performed statistical tests on all models, see Methods. Upper (**a**,**b**) panels correspond to networks with signs created using unidimensional opinions (see legend). For both models $A_1$ and $E_1$, the differences ($p_D-p_M$) are not significantly larger than 0. Thus, the models explain the observed densities of balanced triads without assuming triad dynamics. Middle (**c**–**e**) panels correspond to networks with signs created using multidimensional opinions with different tolerance values (numbers next to the data points). Observed differences ($p_D-p_M$) for the models $A_8$, $E_8$ and $C_8$ are, in general, and for chosen specific tolerances, significantly larger than 0. Therefore, having eight-dimensional triads, random models not assuming Heider Balance interactions cannot explain observed densities of balanced triads. Panels (**f**–**g**) check whether the same conclusion could be reached having a smaller number of attributes *n*. These panels present, among others, the box plots of *p*-values obtained in the statistical analysis to test whether the real curve $p_D(\Theta )$ is comparable to those generated in the corresponding models. The group of *n* attributes comprises statistical tests for all possible combinations. The results show values averaged over six terms and weighted with numbers of triads in each term. Each data point is the result of generating at least 1000 for panels (**a**–**e**) or 100 for panels (**f**–**g**) random models.

The obtained absolute numbers (or densities) of balanced triads do not directly prove that HBT is a significant factor. When a link is labeled by agents’ unidimensional attributes the only unbalanced triad in HBT consists of all negative links. Such a case does not frequently arise for three-state opinions, which is why we expect most triads to be balanced. In the case of multidimensional triads, low tolerances result in most links negative, thus most triads are unbalanced, and vice versa for large $\Theta$ values, most relations are friendly and, therefore, triads are usually balanced. However, the only observable manifestation of the presence of the Heider Balance Theory dynamics is the higher frequency of balanced triads than expected at random. To statistically evaluate whether the numbers of balanced and unbalanced triads do not arise by chance, we proposed three types of alternative statistical models ($A_n$, $E_n$ and $C_n$) where *n* denotes the number of attributes considered, out of which we studied five of them ($A_1$, $E_1$, $A_8$, $E_8$, $C_8$) more extensively (see Fig. 4; the remaining ones are described in SM). These models use the existing network structures but do not regard triadic interactions in the process of formation of attributes and signs.

In model $A_1$, we consider each attribute separately, while in model $E_1$, we consider edges whose signs are based on only one attribute. In the models $A_8$, $E_8$ and $C_8$, we consider all eight attributes together. The models and performed statistical tests are explained in the “Methods” Section. Here, we briefly describe the essential details.

In models $A_n$, we generate new attributes for each agent while keeping the probabilities of $+1$, 0 and $-1$ on a given topic the same as in original data. In this way, we may generate multiple alternative signed networks both with simple ($A_1$) and multidimensional ($A_8$) triads. In models $E_n$, we keep the probabilities to obtain positive and negative edges the same as in networks derived from the real data and, then, we use these probabilities to generate many copies of alternative signed networks. In models $C_n$, again, new attribute sets are generated. Apart from keeping specific opinion probabilities, the correlations between opinions are also preserved.

Figure 4a compares the probability $p_D$ of balanced triads calculated based on the data with the corresponding probability $p_{A_1}$ obtained from the model $A_1$. Statistical tests of the hypothesis that the data can be described using the proposed model resulted in *p*-values larger than 0.05 for all topics, where the smallest one equals 0.079. Thus, the model $A_1$ cannot be rejected. In other words, the observed statistics of simple triads related to singular attributes do not indicate Heider interactions since they can be explained by a random distribution of opinions among the students.

For the model $E_1$, the statistical test resulted in *p*-values larger than 0.05 for all topics except gay marriage (0.0079) (see Fig. 4b). However, we cannot reject the model for any of the opinions by controlling the family-wise error rate using the Holm-Bonferroni method at the significance level of 0.05. In other words, the observed statistics of simple triads related to singular attributes do not indicate Heider interactions since a random arrangement of links between students can explain them.

Figure 4c compares the probability $p_D$ of balanced triads calculated based on the data with the probability $p_{A_8}$ calculated based on the model $A_8$. Here, a two-level test was performed. The probability of obtaining a sum of ranks not lower than for the real $p_D(\Theta )$ was 0.038. Therefore, the hypothesis that the real curve $p_D(\Theta )$ is like those obtained in the model $p_{A_8}(\Theta )$ was rejected. Then, for each specific $\Theta$, we performed one-side tests, which allowed us to conclude that the probability of balanced triads is larger in data for $\Theta \le 0.5$. *P*-values calculated for this model are below 0.001 for $\Theta <0.5$ and equal to 0.002 for $\Theta =0.5$. It means that assuming small or medium tolerances in the link sign definition, we can observe the effects of Heider interactions because the observed high densities of balanced triads are not explained with the model accounting only for dyadic interactions.

For model $E_8$, the probability of balanced triads in data is significantly larger for tolerances 0 and in the range [0.1875, 0.8125] (Fig. 4d). However, keeping the family-wise error rate at the significance level of 0.05 would obtain a smaller range of $\Theta \in [0.1875, 0.625]\cup 0.75$ with all *p*-values below 0.01. Thus, this model explains high densities of balanced triads for the lowest tolerances, but high densities are unlikely to arise by chance for the medium values.

Figure 4e compares the densities of balanced triads in the real data ($p_D$) and in the model ($p_{C_8}$). Again, as tests for specific tolerances are not independent, we performed two-level tests. The real curve lies significantly above curves from the model with a *p*-value equal to 0.005. Comparing specific tolerances, the densities of balanced triads were higher for most tolerances, but significant results were obtained only for $\Theta \in \{0, 0.1875, 0.8125\}$ with respective *p*-values of 0.028, 0.017 and 0.023. Thus, if taking most of the specific tolerance values, it might seem that Heider interactions are not observed. But at the same time, the model cannot explain the high densities of balanced triads observed for all the tolerances. It means that for a given $\Theta$, it is not unlikely that random data sets will give as high densities of balanced triads as those measured in the real data set. However, considering the whole relation $p_{C_8}(\Theta )$, we must reject the hypothesis that the model explains the results for the entire data set.

For the models of multidimensional triads ($A_8$, $E_8$, $C_8$), the obtained real curve of relation $p_M(\Theta )$ (where *M* is the given model) lies significantly lower than for the real data set. It implies that the models accounting only for dyadic interactions do not explain the results from the real data set and imply that observed high densities of balanced triads are caused by interactions of order higher than dyadic, including Heider interactions. Looking for specific tolerance values, we found only one such value ($\Theta =0.1875$) for which the probability of balanced triads is always significantly larger in the real data. Comparing panels (a–e) in Fig. 4, we see there is about 64% ($A_8$), 32% ($E_8$) and 28% ($C_8$) less balanced triads in model (for maximal point) than in the data, while the corresponding numbers for single opinion models are 5% ($A_1$) and 8% ($E_1$). This observation, combined with the results of our statistical tests, indicates that while it is improbable to notice Heider interactions using models with a singular attribute, they are likely to be observed for triads built in our eight-dimensional model.

Observation of Heider interactions in the analyzed dataset requires multidimensional opinions. To determine how many opinions are necessary, we performed statistical analysis for each subset of *n* opinions for models $A_n$, $E_n$ and $C_n$ with *n* varying from 1 to 8. Full results are presented in SM. Panels (f–g) of Fig. 4 show the *p*-values of first-level tests for models $A_n$ and $C_n$ (whether the real curve $p_D(\Theta )$ is comparable to those generated in corresponding models). The panels show both the box plots of *p*-values obtained for different combinations of attributes and the ratio indicating for how many combinations the hypothesis was rejected. For instance, if the dataset consisted of only four attributes, we might still notice Heider interactions, but our conclusions would depend on the specific choice of topics. The first level hypothesis would be rejected in the case of 64% ($A_4$) or 47% ($C_4$) of all choices. Overall, we may conclude that having almost any combination of 7 attributes would allow observation of Heider interactions.

### Dynamical properties of triads

Heider Balance Theory also postulates that balanced triads are more stable than unbalanced ones. A natural way to measure a triad’s persistence is to count the number of terms in which this triad exists. However, the experiment lasted just six terms and created only a small network. Thus, such a measure could only yield insignificant results. Hence, we propose another measure—*transition probability for triads*, which is the probability of a change from one type of a triad to another between study terms. The proposed measure does not depend on the length of data.

**Figure 5:**
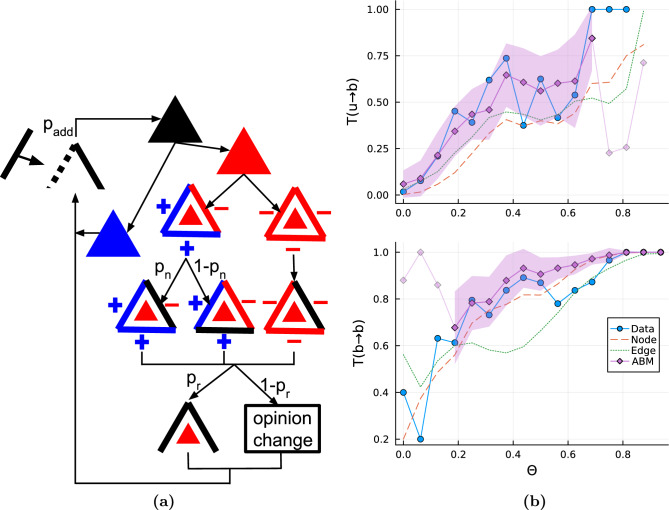
(**a**) Decision tree of the dynamics of the proposed agent-based Heider Balance model. Our model algorithm processes the network iteratively in update steps. First, a random triad ($\blacktriangle$) is selected. It can either be balanced () or unbalanced (). If the triad is unbalanced, we select its edge (drawn in black in the figure). For the triad with two positive edges, the chosen edge is positive or negative with probabilities $(1-p_n)$ or $p_n$, respectively. For the triad with three negative edges, we choose one of them randomly. Then, we either remove the selected edge with probability $p_r$ or change an opinion of one of the edge endpoints with probability $(1-p_r)$ so that changing the edge’s sign will be possible. For a full description, see the main text. At the end of the single update with probability $p_{add}$, a new edge is added to the graph. (**b**) Transition probabilities in the NetSense data lie between the results of the agent-based model with HBT dynamics and of the random models. Plots show transition probabilities from unbalanced $T({u\rightarrow b})$ or balanced $T({b\rightarrow b})$) triads to balanced ones as a function of the tolerance $\Theta$. The solid blue lines with circles represent empirical results. Dashed red and dotted green lines show results for node- and edge-randomized models, respectively. Solid purple lines with diamonds show the results of our agent-based model (based on diagram 5a). Fuzzy markers are drawn where too few triads of a given type make the obtained probabilities unreliable. Results imply that the dynamics of triads recorded in the empirical data set are driven by both Heider interactions and processes generating randomized data. Each model data point was obtained by at least 100 simulation realizations. The shaded area shows standard deviations of ABM transition probabilities.

As expected, probabilities of transition from unbalanced-to-balanced $T({u\rightarrow b})$ and from balanced-to-balanced triads $T({b\rightarrow b})$ (shown in Fig. 5b) tend to increase with tolerance. Note that other transitions ($T({b\rightarrow u})$ and $T({u\rightarrow u})$) are complementary, therefore they are not presented. We introduced two types of randomizations to compare reliably computational results with the observations obtained from the real data. The first is a *node-randomized* model that preserves the flipping rate of the smallest possible element, that is, an agent’s opinion. The second type is an *edge-randomized* model that works similarly, except for flipping rates of edge signs (from positive to negative and opposite) that, in this case, were equal to those observed in the NetSense data. Results obtained from random models also show an increase of both probabilities, $T({u\rightarrow b})$ and $T({b\rightarrow b})$, with the growing tolerance. The results of $T({b\rightarrow b})$ for the real data and node-randomized networks are similar. However, for both random models, the probabilities $T({u\rightarrow b})$ are significantly lower than the empirical ones. Students change their opinions in response to many processes unknown to us. The effects of these processes can be recreated using the proposed models. The results show that these models are good at generating changes giving similar transition probabilities $T({b\rightarrow b})$. However, they are insufficient for the transitions from unbalanced triads to balanced ones. Thus, the rational conclusion is that the mechanisms of triads’ dynamics postulated by Heider Balance Theory generate the observed results. In other words, changes of students’ opinions and acquaintances lead to the emergence of balanced triads.

Based on these conclusions, we propose an agent-based data-driven model of evolving social group behavior. The model extends previous models introduced in^20,28^. The model’s initial state is based on the first term of the NetSense data set; that is, the number of agents, exact links and agents’ attributes $\sigma ^i_t$ are determined. In every step of the model, we perform the following actions (also shown in the decision tree in Fig. 5a):

1. A random triad (*ijk*) connecting agents *i*, *j* and *k* is chosen.
2. If the triad is balanced, nothing happens, and step 6 follows. Otherwise, in the next step, we choose one triad’s link.
3. If there are two positive edges in the triad, then with probability $p_n$, we choose the negative edge. Otherwise, with equal probability $(1-p_n)/2$, we randomly select one of two positive edges. If all the triad’s edges are negative, with equal probability 1/3, we randomly choose one of them and denote it (*ij*).
4. Then, with probabilities $p_r$ and $(1-p_r)$, the selected edge is either removed or an opinion of one of its endpoints is adjusted (see the next step).
5. Having decided to change the opinion for one of the agents (e.g., *i*), we choose a topic *t*, which can change the sign of edge (*ij*). That is, the opinion is randomly chosen from the set of topics such that the change of this opinion makes *i* and *j* more similar when the edge (*ij*) is negative and less similar otherwise. And then $\sigma _t^i$ changes by the smallest distance increment closer or further to the other agent’s opinion.
6. A new edge is added randomly to the graph with the constant probability $p_{add}$.
7. Step 1 follows until the predefined number of iterations is reached.

Although the model directly applies the concept of unbalanced triad evolution, the model is reasonable because, with each intentional change, plenty of accidental changes are possible (see^28^), which may cause the transitions from balanced to unbalanced structures. The model has three basic parameters: $p_n$—probability of trying to change the negative link in triads with one negative edge, $p_r$—ratio of choosing edge removal over opinion update while trying to modify edge sign, and $p_{add}$—probability of adding an edge in a single update.

Further, we calibrated the probability $p_n$ using the real data and obtained the rest of the parameters by performing a set of simulations in a validation procedure (see SM for details). The specific values of obtained parameters are as follows: $p_n=0.4$, $p_r=0.15$, $p_{add}=0.07$.

Figure 5b compares the model simulation results with the results obtained from the real data and two random models. First, we see a difference between the agent-based model (ABM) and the real results for the three smallest tolerances in the case of $T({b\rightarrow b})$ and largest tolerances ($\Theta \ge 0.75$) in the case of $T({u\rightarrow b})$. This is caused by the small number of respective triads in the system (balanced for $T({b\rightarrow b})$ and unbalanced for $T({u\rightarrow b})$). For instance, for $\Theta =0$, there are only two balanced triads, out of which one is not connected to other triads; therefore, if no new triads are created, this triad won’t change and as a result, $T({b\rightarrow b})$ is between 0.5 and 1. The majority of balanced triads can be affected by the dynamics for $\Theta >0.125$. Similarly, for the largest tolerances, the numbers of unbalanced triads are very small, and the ABM dynamics can change the system for $\Theta <0.75$. The following discussion concerns only those ranges. Plots of transition probabilities for the model and real data follow similar trajectories. For $T({b\rightarrow b})$, the results of ABM and node-randomization give values comparably close to the real probabilities. The big difference is for $T({u\rightarrow b})$, where the ABM results are much closer to the results from the real data set for tolerance values up to $\Theta =0.4$. In the range [0.4, 0.7], the ABM results are the upper bound of the NetSense transition rates. In other words, our analysis of the behavior of social groups recorded in the NetSense experiment reveals the agents’ preference for belonging to the balanced rather than unbalanced triads as postulated by the Heider Balance Theory. The model results are closest to the real data for intermediate values of the tolerance $\Theta$ ($\sim 0.15<\Theta <0.4$), which are the best values to observe Heider interactions. For small values of $\Theta$, the methodology is not sensitive enough since most links are classified as negative. For the range ($\sim 0.4<\Theta <0.7$), triad transitions for the real data lie between the ABM and randomized networks. For large values of $\Theta$, most links are classified as positive, causing all transition probabilities to tend to one, so it is not possible to make any reliable conclusions. The most important conclusion from these numerical experiments is that the similarity of behaviors between simulations and the real data confirms the existence of the triad interactions postulated by the Heider Balance Theory defined over the range of tolerance parameters in the case of multidimensional space of attributes.

## Discussion

Understanding the processes of link creation and opinion formation is crucial for human behavior modeling. Higher-order interactions (e.g., triadic or higher order) can influence these processes^18,55^. Here, we present the role of multidimensional social attributes for triadic interactions in the student community. We consider multiple students’ opinions on eight topics together to get an integrated view of this social system. We show that static and dynamic properties of triadic interactions observed in our study confirm the multidimensional character of attributes in Heider Balance Theory. To label social links with such attributes, we use the concept of the Manhattan distance in multidimensional opinion space and study the stability of social triads for various values of the tolerance parameter $\Theta$ that enables the classification of social links to be positive or negative.

By using agents’ opinions to derive edge signs and by analyzing the balance of obtained signed triads, we combine dyadic and triadic interactions. Dyadic interactions, such as homophily, are often analyzed together with triadic measures like triadic closure^9,11^. Triadic closure can be responsible to some extent for the over-representation of triads with three positive links. Although triads in the NetSense data set are over-represented (see SM, Table S3), in this paper, we did not analyze this excess but took it as a fact. Here, we focus on finding what kinds of signed networks created from our data will have more balanced triads than expected at random.

Figure 4 demonstrates that the number of balanced triads in the NetSense data defined in eight-dimensional space is significantly larger than the corresponding numbers predicted from statistical models $A_8$, $E_8$, $C_8$ that consider eight social attributes together and neglect Heider interactions. On the other hand, one-dimensional models $A_1$ and $E_1$ that apply singular attributes separately generate the numbers of balanced triads comparable to those observed for single opinions in the NetSense data set. With unidimensional attributes, unbalanced triads are rare, causing the sensitivity of determining whether Heider Balance Theory is a significant factor to be very low since the observed patterns are close to the results from null models when agent attributes or social links are randomly distributed. It means that Heider interactions are either difficult to measure or negligible for singular students’ opinions (e.g., attitudes towards abortion, death penalty). Yet, they are significant when interactions are defined over multidimensional vectors of attributes. This agrees with the discussion in the study^56^.

We also found that the effect of social balance can be observed for low or intermediate values of the tolerance parameter $\Theta$ (approximately $0.15< \Theta <0.4$). The parameter $\Theta$ corresponds to the level of sensitivity for the classification of social links to be positive or negative. For this parameter’s extreme values, many dyads are labeled as negative (when $\Theta$ is very low) or positive (when $\Theta$ is close to one). It follows that such extreme values of $\Theta$ do not provide enough information for the subtle effects of HBT interactions to be observable. Application of the obtained results for other systems is, therefore, dependent on the tolerance value agents in the given system have. If the tolerance is measured, then our results will tell whether one may expect to observe Heider interactions easily or they will be hard to observe. In the former case (for tolerances $\sim 0.15<\Theta <0.4$), not observing Heider interactions would be a significant result against the importance of these processes in the given system. In the latter case, a very small or large tolerance value may be the reason for not finding significant proof for the Heider Balance Theory. This may be another reason for not finding Heider dynamics as a significant factor for forming relations in some of the data sets^15,16^. Since individual declarations of friendships may depend on individual values of $\Theta$, HBT could still be one of the underlying processes. Yet, when people tend to create positive links (true or virtual) easily with people who are not very similar to them, then HBT is challenging to observe.

In the above paragraphs, we mentioned that to observe Heider interactions, one needs to have a sufficient level of sensitivity, especially when the signs of the links are defined but not given. In the analyzed data set, the level of sensitivity is low when having singular opinions and high when eight-dimensional attributes are considered. If the data set consisted of a lower number of topics, the obtained level of sensitivity would depend on the choice of topics. Having high-dimensional attributes is just a sufficient condition since we do not know if multidimensional attributes are always required. We also hypothesize that not only attributes’ multidimensionality exposes Heider balance dynamics to measurements but also high attribute granularity. We plan to verify this hypothesis in future work.

The need for the presence of Heider interactions in multidimensional space is also confirmed by the analysis of the dynamical properties of the same data set. In the framework of our agent-based model, unbalanced triads can change intentionally introducing at the same time accidental changes in the rest of the network. Results presented in Fig. 5b show that the transition from balanced states is well described by random processes (randomized networks or accidental changes in the agent-based model). However, random processes are not enough to explain the transition from unbalanced to balanced states. In this case, the agent-based model with Heider interactions yields results much closer to reality than to the random models. Probabilities of this transition as functions of tolerances for the Manhattan distance for the NetSense data are larger than the ones obtained from random models. It means that students tend to build balanced triads in the eight-dimensional attributes space by changing their social links and opinions more frequently than it could have been caused just by independent changes of their opinions or by homophilic dyadic interactions. In fact, recently, it has been postulated that homophilic dyadic relations are enough to explain in another data set the observed statistics of balanced and unbalanced triads^52^. Our tests on the NetSense data set still show that the proposed ABM model gives closer results to the real ones than the model based on dyadic relations (see SM). However, when only Heider interactions in the eight-dimensional space of opinions are considered, as in the agent-based model, then transition rates from balanced-to-balanced triads are slightly higher than transition rates in the observed data. It means that social dynamics are a mixture of random opinion flipping, changes resulting from homophilic dyadic relations and Heider interactions based on multidimensional attributes.

People can change their social networks by adding new friends or removing old ones. To reduce tensions^2^ and achieve structural balance, they can also change their opinions. Here, we see that the multidimensional character of human opinions enables the model to replace crude flipping of a sign of human relations (from friendship to enmity and vice versa)^20^ with a subtle change of a single attribute^28^. Our approach can analyze the influence of different parameters on system behavior. We found a good agreement between the model and data when the probability of changing a negative link (in an unbalanced triad with one negative link, see Fig. 5a) towards a positive one at $p_n=0.4$. This value indicates that preference towards forming a positive or negative relation is similar, agents agree to have negative links and the expected outcome of the system is a mixed state of positive and negative edges^28^.

In models considered in^20,28^, triads’ states evolved only by changing existing edge or node states. However, removing and adding edges also drives the evolution of social networks^10,12,15^. In our approach, the link rewiring observed in real data is defined by parameters $p_r$ and $p_{add}$; thus, the proposed model merges HBT with the co-evolution of social networks^10,12,41,42,57^.

Obtained results give insight into modeling opinion dynamics with the usage of triadic interactions. The presented research uncovers the importance of multidimensionality (understood as the multiplicity of social attributes describing a given person) in real-world scenarios. Observed interactions and dependencies can help in developing better understanding of emerging social systems, like new student classes in school, new departments in companies or new sports activity groups. Analysis of inside group processes can guide enrolling new group members to increase efficiency and to improve relations and social atmosphere^12,56^.

## Methods

### Simple and multidimensional triads

In the case of simple triads, an edge sign is defined using a distance between opinions on a specific topic. If both connected students hold the same opinion on the topic, the edge is positive and otherwise—negative. One can also consider a different way of defining edge signs: an edge is positive if it connects two nodes with the same or similar opinion (*unsure*–*in support* and *unsure*–*against*) and negative if it connects agents with two extremist opinions (*in support*–*against*). In both edge sign definitions, one will get the same types of triads’ balance (see Fig. 1). Therefore, in the presented analysis, the first interpretation is used.

Using the approach of simple triads, each of the topics allows us to define a different edge sign. Hence, one network per topic is obtained. Here, we are interested in analyzing the mutual influence of opinions and interactions, and the above approach allows us to study each topic separately. As a result, some of the dependencies may stay unnoticed. Therefore, we also defined multi-edge signs that consider the distances between opinions on all topics in the sign definition. In general, the definition of the multi-edge sign $s^{i,j}$ connecting agents *i* and *j* is given by $s^{i,j}=f\big ( d^{i,j} \big )$, where $d^{i,j}$ is the distance between agents in the multidimensional attribute space and *f* is the function translating the distance into the specific relation: positive or negative.

Here, we applied the Manhattan metric as the distance between agents, creating a measure similar to Gower similarity^58^. In other approaches^38,39,51,54,59^ Euclidean distance, cosine similarity or other functions were also used. Let $\sigma ^i_t \in \{-1,0,1\}$ be an opinion of a student *i* on a topic $t=1,2,\ldots , n$ (in our case $n=8$) and $\Delta ^{i,j}_t= | \sigma ^i_t - \sigma ^j_t|$ be the difference in *t*-th opinion between students *i* and *j*. A normalized distance $x^{i,j}$ between students is defined as the Manhattan distance $x^{i,j}= \frac{1}{2n}\sum _{t=1}^n \Delta ^{i,j}_t$. Two extremists with opposing opinions on all topics will have the normalized distance 1. Two students with identical opinions will have a distance 0. The smallest increment of the distance is $\frac{1}{2n}$, e.g., when the neutral opinion about one topic of one of the students changes to extremist. Further, to obtain edge signs, we applied a tolerance function. The multi-edge between students (*i*, *j*) is positive, when $x^{i,j} \le \Theta$, where $\Theta$ is the selected value of tolerance ($0\le \Theta \le 1$). Our way of creating multi-edge signs is by binarization of a distance metric. Ref.^39^ used for that purpose a sigmoidal function. The drawback of our approach is that the information about the distribution of distance metric is lost. However, the positive side is that signed networks are obtained, and the Heider Balance Theory may be studied.

Sign definitions in simple and multidimensional triads lead to different observed triad types. In the case of simple triads, as shown in Fig. 1, only one type of unbalanced triad is feasible (regardless of the chosen interpretation). The triad with one negative link is unachievable. In the case of multi-edge triads, in general, for *n* topics, all types of triads are possible (i.e., with 0, 1, 2, or 3 negative links).

We treat all topics uniformly. Equal distances between opinions are approximations since often some opinions are more fundamental to extremists than others. Yet, attempting to tune the distances with the data we have available would likely lead to overfitting. Moreover, Manhattan distances ignore correlations between opinions that could affect the structure of the obtained signed network. Therefore, we have also created multi-edges after the correlations between individual opinions have been removed. See SM, sections “Opinion correlation” and “Static properties of triads for multi-edges without correlations” for more details. The obtained results and conclusions for the approaches based on such metrics stayed the same. Thus, in this text, we use only the distance definition described in the previous paragraphs.

Our procedure of evaluating the expectations of HBT in the data-driven network is subject to network sampling error because we estimate balance only among triplets of agents that communicated with each other. Although contact avoidance could be caused by the enmity among students (i.e., negative relations), it is, however, indistinguishable from other factors (e.g., not knowing each other or using other means of communication). The sampling error is minimized because the community of analyzed students are classmates, and most of them do not know each other when arriving at the university since the University of Notre Dame tries to avoid accepting more than one student from the same high school (see also Fig. S1 in the SM showing little differences between similarity distributions among communicating agents and the whole network).

### Random models of signed networks

Balanced triads should be more frequent than expected at random if processes resulting from the Heider Balance Theory are significant and observable. Therefore, in the first subsection of the Results, we compared the real densities of balanced triads with the ones obtained in random models.

No probabilities in the given term were computed for agents who did not form any connections during that term. For each model, we calculated *p*-values for one-tailed permutation tests whether the models can explain the observed density of balanced triads $p_D$^60^. To analyze models $A_1$, $E_1$ or $E_8$, a test was made for each attribute (or each considered tolerance value) separately, and the Holm-Bonferroni method was applied to control the family-wise error rate at the significance level of 0.05. When considering multidimensional triads in models $A_8$ and $C_8$, the densities $p_{D}(\Theta )$, $p_{A_8}(\Theta )$ and $p_{C_8}(\Theta )$ for different tolerances are not independent from each other. This is because in these models, we first generate a random data set and then we calculate the relation $p_{M}(\Theta )$ for each data set (where *M* is the given model). One can still perform statistical tests for each tolerance value, but these tests are correlated. Therefore, we used a two-level testing scheme. First, we determined whether the relation $p_{D}(\Theta )$ gives significantly larger values than relations $p_{M}(\Theta )$. This was performed by ranking the densities for specific $\Theta$, then summing up the ranks for each data set, and finally calculating the probability of obtaining such a sum for relation $p_{D}(\Theta )$ in randomized data. Secondly, we found specific values of $\Theta$ for which $p_{D}(\Theta ) > p_M(\Theta )$ by performing permutation tests. The testing scheme is described in SM in more detail.

In the first model ($A_1$), we consider simple triads and, when generating new attributes, we do not assume any type of influence (neither between different attributes nor between connected agents). Opinions in the model are generated with the corresponding probabilities from the real data. Let *q*, *r* and *s* $(q+r+s=1)$ be probabilities of finding the opinion $+1$, 0 and $-1$ on a given topic (e.g., marijuana), respectively. If opinions of agents in a network are uncorrelated, then a triad is balanced with a probability $p_{A_1}=q^3+r^3+s^3 +3q^2(r+s)+3r^2(q+s)+3s^2(q+r)$. After reduction $p_{A_1}=1-6qrs$. In the case of a two-state system (i.e., when either $q=0$ or $r=0$ or $s=0$), all triads are balanced.

The second model ($E_1$) assumes homophily is the only relation driving evolution. Here, the signs are generated with the corresponding probabilities from the real data. Let *a* be the probability that a link between two agents is positive (there is an agreement on a given topic) and *b* be the probability that the link is negative $(a+b=1)$. It is easy to conclude that with opinions defining dyadic relations for a single topic, only three kinds of triads in terms of link relations are possible since a triad with one negative and two positive edges is not feasible. In the model, we randomly distribute links considering this conclusion. For instance, having an unclosed triad of two positive links, we know the third one must be positive. Then the random triad is balanced with a probability $p_{E_1}=1-b^3$.

The third model ($A_8$) considers multidimensional triads under the assumption of no correlations between opinions about eight different topics by the same agent and no correlations between opinions of different agents. Here, opinions are generated similarly as in model $A_1$. Let $q_t, r_t, s_t$ be the probabilities of opinions $-1, 0, 1$ of agent *i* on topic $t=1,2,\ldots , 8,$ respectively. The probability distribution $p_t(\Delta _t)$ can be calculated as functions of these probabilities. For example, $p_t(\Delta _t=0)= q_t^2+ r_t^2+s_t^2$ and $p_t(\Delta _t=1)$, $p_t(\Delta _t=2)$ can be obtained analogically. The probability distribution of distances $P_{8}(x)$ is eight-times convolution of all $p_t$’s. Considering the observed values of $q_t, r_t, s_t$ for different topics, we get the distribution of $P_8(x)$. Following the definition of the positive link between agents (*i*, *j*), its probability being positive is $a(\Theta )=\sum _{x=0}^\Theta P_8(x)$. Hence the probability of getting a balanced multidimensional triad is $p_{A_8}(\Theta )=a(\Theta )^3+3a(\Theta )b(\Theta )^2$ where $b(\Theta )=1-a(\Theta )$.

The fourth model ($E_8$) also considers multidimensional triads but with assumptions that homophily drives evolution. This model is an extension of the model $E_1$. Again, we consider randomly distributed signs of links with probabilities *a* for a positive link and $b=1-a$ for a negative one. For multidimensional triads, all kinds of triads are possible, yet the probability of obtaining a balanced triad is simple, as $p_{E_8}(\Theta )=a(\Theta )^3+3a(\Theta )b(\Theta )^2$.

The fifth model ($C_8$) assumes that different opinions are correlated, but it disregards correlations among agents. Each random data set is created by shuffling students’ real sets of opinions.

Analysis for models with intermediate numbers of attributes $A_n$, $E_n$ and $C_n$ was performed in the following way. For each number of attributes *n*, for each combination of *n* attributes, we generated random data sets and performed similar one- or two-level tests as described above.

### Analysis of triads’ transitions

#### Measuring transition probabilities

To measure the probabilities of triads’ transitions, we enumerated the number of changes of each type between study terms. From term to term, each triad could stay in its state (balanced or unbalanced), change to the opposite state or dissolve. The fraction of disappearing triads was high due to the decreasing number of links in the social network. The disappearance of the links was caused mostly by unrelated to this study link removal from the data set (e.g., by students dropping from classes, transferring to other universities or by students changing means of communication), in addition to students’ social interactions. Therefore, we did not discuss the triad dissolution probability, even though Heider Balance Theory expects balanced triads to decay slower and it is true for our data set (see SM, Fig. S6).

#### Randomizations

In the *node-randomized* model, we changed agents’ opinions with probabilities obtained from the NetSense data. The probabilities were different for each topic. The initial opinions were the same as in the real data and we allowed all opinions to change four times to enable a transition from one extreme opinion to the opposite one. Then, we measured probabilities of triad changes from data with randomized node states. In the *edge-randomized* model, we flipped the signs of edges (from positive to negative and opposite) with probabilities equal to those observed in the NetSense data. In this case, the signs changed in one-step.

### Supplementary Information

Supplementary Information.

## Data Availability

The datasets generated during and/or analyzed during the current study are available from the authors on reasonable request.

## Code availability

Source code related to agent-based model, as well as scripts that read the data, perform data analysis, and generate random models are available at https://github.com/pjgorski/NetHeider.
