# Supplementary material for: Multidimensional attributes expose Heider balance dynamics to measurements
Source: Sci Rep. 2023 Sep 20;13:15568. doi: 10.1038/s41598-023-42390-w (PMC10511462; doi:10.1038/s41598-023-42390-w)
Supplement: Supplementary file 1 — Supplementary Information. [file 41598_2023_42390_MOESM1_ESM.pdf]

Supplementary Materials for

Multidimensional attributes expose Heider balance dynamics to measurements

Joanna Linczuk[1,+], **Piotr J. Górski**[1,+,*], **Boleslaw K. Szymanski**[2,3,*], **and Janusz A. Hołyst**[1]

[1]Faculty of Physics, Warsaw University of Technology, Koszykowa 75, 00-662 Warszawa, Poland
[2]NEST Center, Dept. Computer Science, Rensselaer Polytechnic Institute, 110 8th Street, Troy, NY 12180-3590, USA
[3]Academy of Social Sciences, Henryka Sienkiewicza 9, 90-113 Łódź, Poland
[*]E-mails: piotr.gorski@pw.edu.pl, szymab@rpi.com.
[+]These authors contributed equally to this work.

This document presents supplementary material for the paper entitled "Multidimensional attributes expose triad dynamics to measurements". Section S1 describes the coding scheme used to obtain $\{-1, 0, +1\}$ attribute values from students' questionnaires. Section S2 shows distributions of the density of distances between agents in the attribute space, explains observed correlations between agents, and introduces the method for removing the correlations. Section S3 describes the obtained signed network and presents some statistics related to observed balanced and unbalanced triads. Section S4 describes the statistical method used to compare the real network and random models. Section S5 shows the results for the multi-edges with correlations removed. In section S6, we compare the real and randomized networks in the case of intermediate numbers of attributes considered. Section S7 describes the proposed agent-based model (ABM) in more detail, introduces an error function used to fit the model to data, and compares different models. This section also describes another ABM model that assumes dyadic interactions only.

S1 Coding of students' opinions

We used the same scheme as in[1] to obtain students' opinions coded as *Being against*/*Unsure*/*In support* labels. The coding system is shown in Tab. S1. As shown in the table, usually *Unsure* label is just a "Not sure" answer. It may also signify a student "neither agreeing nor disagreeing" in the case of Gay marriage or a student with "Moderate" political alignment. That is why Ref.[1] named this column either as *Unsure* or *Neutral*.

S2 Distance distributions and opinion correlation

Figures S1a and S1b present distributions of Manhattan distances between eight-dimensional attributes of connected students and all pairs of students, respectively. The two sets of samples are not very different. However, as it was verified with the Kolmogorov-Smirnov test giving a p-value of 0.020, they were drawn from distinct probability distributions. The major difference is related to the larger proportion of very similar pairs (distance around zero) for a real data set.

S2.1 Opinion correlation

If opinions were fully correlated, two groups of agents would have formed, for instance, groups with more liberal or more conservative views on topics. In such a case, the distance distribution would contain two peaks: one for lower distance (i.e., people that agree on their worldview) and one for larger distance (i.e., people with different worldviews). As is shown in Fig. S1, this is not the case. However, the observed discrepancy in the density of most similar pairs in real and random data sets indicates that correlations might play some role. Therefore, we measured the Spearman rank correlation coefficients between the considered topics. The results for the first term questionnaire are presented in Tab. S2.

The results confirm that some of the opinions are correlated. Therefore, we created multi-edges with correlations removed. For the opinion vectors from term 1, we applied Principal Component Analysis (PCA) and transformed the opinion values for the new coordination system (indicated with *hats* over variables in the equation below). The distance measure stays the same,

Topic	Being against	Unsure/Neutral	In support
Abortion	• By law, abortion should never be permitted<br>• Law should permit abortion only in case of rape, incest, or when the woman's life is in danger	• Not sure	• Law should permit abortion in other cases, but only after the need for the abortion has been established<br>• By law, a woman should always be able to obtain an abortion
Death penalty	• Oppose	• Not sure	• Favor
Euthanasia	• No	• Not sure	• Yes
Gay marriage	• Strongly disagree<br>• Disagree	• Neither agree nor disagree<br>• Not Sure	• Agree<br>• Strongly agree
Homosexuality	• Always wrong<br>• Almost always wrong	• Not sure	• Sometimes wrong<br>• Not wrong at all
Marijuana	• Not Legal	• Not Sure	• Legal
Political	• Extremely conservative<br>• Conservative<br>• Slightly conservative	• Moderate<br>• Not sure	• Slightly liberal<br>• Liberal<br>• Extremely liberal
Premaritalsex	• Always wrong<br>• Almost always wrong	• Not sure	• Sometimes wrong<br>• Not wrong at all

Table S1. The coding system for the considered topics.

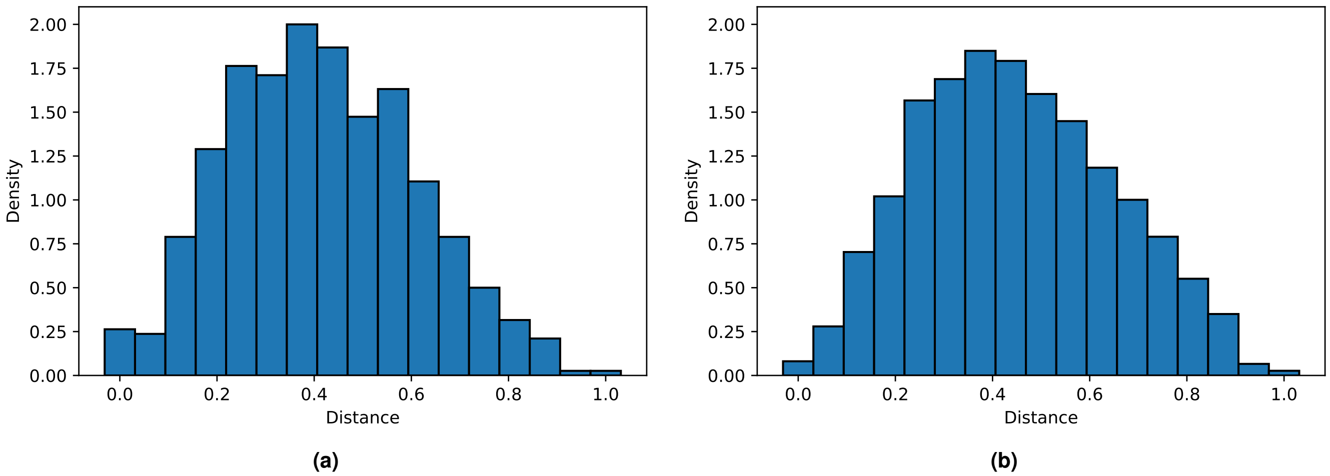

Figure S1. Comparison between Manhattan distance distribution among connected (a) and all pairs of agents (b). The panels show the density of pairs of students' distances.

	Opinion	1	2	3	4	5	6	7	8
1	**Abortion**	1.000	0.289	0.335	0.477	0.631	0.315	0.474	0.473
2	**Death penalty**	0.289	1.000	0.173	0.098	0.154	0.067	0.032	0.298
3	**Euthanasia**	0.335	0.173	1.000	0.290	0.333	0.261	0.145	0.369
4	**Gay marriage**	0.477	0.098	0.290	1.000	0.709	0.288	0.424	0.463
5	**Homosexuality**	0.631	0.154	0.333	0.709	1.000	0.341	0.460	0.622
6	**Marijuana**	0.315	0.067	0.261	0.288	0.341	1.000	0.394	0.310
7	**Premarital sex**	0.474	0.032	0.145	0.424	0.460	0.394	1.000	0.406
8	**Political**	0.473	0.298	0.369	0.463	0.622	0.310	0.406	1.000

Table S2. **The Spearman rank correlation coefficients for original opinions in the 1st term.**

but now, it is computed as the average value of the sum of absolute values of differences for all attributes and normalized by the new maximum distance (C).

$$\hat{x}^{i,j} = \frac{1}{n}\frac{1}{C}\sum_{t=1}^{n=8}\hat{\Delta}_t^{i,j}, \tag{S1}$$

where $\hat{\Delta}_t^{i,j}$ is an absolute value of differences between t-th principal component of agents i and j, and $C = \max_{i,j}\sum_t \hat{\Delta}_t^{i,j}$ is the normalization constant such that the considered tolerances Θ vary from 0 to 1. The distance distribution after this transformation is presented in Fig. S2.

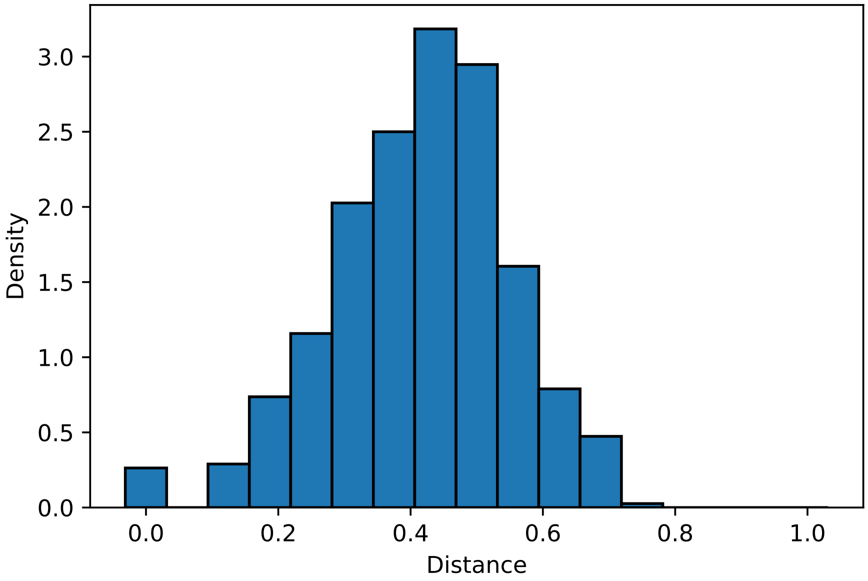

Figure S2. **Distribution of Manhattan distances after transforming opinions using PCA.**

Further results for multi-edges without correlations are shown in the section S5.

S3 Generated signed networks—overview and statistics

Signed networks generated for multi-edges are dependent on the tolerance Θ. Fig. S3 presents the network for the first term for three different tolerance values. As expected, for $\Theta = 0$, there are only two positive links and most triads are unbalanced.

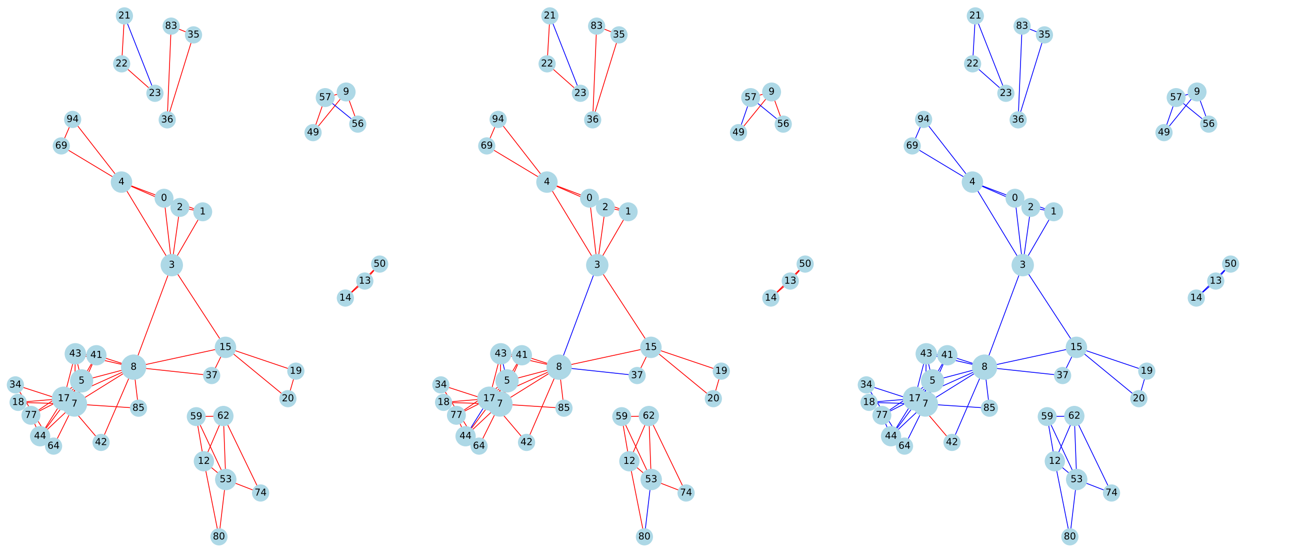

(a) $\Theta = 0$ **(b)** $\Theta = 0.1875$ **(c)** $\Theta = 0.75$

Figure S3. Signed networks with signs generated from multidimensional opinions based on tolerance Θ. The panels show the network from the first term. Only nodes that are part of triads are shown. The network contains six components consisting of 24, 6, 4, 3, 3 and 3 nodes. Blue and red edges correspond to positive and negative relations, respectively.

The largest component starts having both balanced and unbalanced triads from $\Theta = 0.1875$. If $\Theta \geq 0.75$, there is at most one negative link in the network.

The numbers of triads in each term are listed in Fig. 3 in the main text. These numbers are listed explicitly in Tab. S3. This table also lists the number of edges, graph density, and the global and local clustering coefficients.

Term	N	E	# triads	d	T	C_1	C_2
1	98	152	45	0.032	0.22	0.17	0.19
2	89	116	26	0.027	0.26	0.20	0.24
3	68	61	9	0.018	0.33	0.14	0.22
4	89	109	26	0.025	0.27	0.24	0.29
5	85	101	20	0.024	0.24	0.17	0.21
6	63	69	12	0.023	0.25	0.14	0.23

Table S3. Elementary statistics of communication networks from each term. Columns represent: N – number of students actively communicating in each term, E – number of created edges, d – graph connection density, T – transitivity, C_1 and C_2 – measures of average local clustering coefficient. Graph density d and clustering coefficient C_2 consider active students only. The parameter C_1 takes the average over all the students (i.e., 108) included in the analysis.

Considering all the triads from all the terms, the exact numbers of balanced and unbalanced triads $p_D(\Theta)$ are shown in Fig. S4 and Fig. S5. Fig. S4a shows the results in the case of simple triads for different topics, whereas Fig. S5a presents the change of numbers of triads depending on the tolerance value. B-panels of those figures also show histograms of specific triad types.

In the case of simple triads, most triads are balanced. This is not surprising. Unbalanced triads require three nodes to have -1, 0 and $+1$ opinions, which did not often happen. This is also why simple triads are insufficient to observe triad dynamics. Fig. S4b, as defined, does not show triads with one negative edge. Most of the balanced triads are triads with two negative edges.

Fig. 5b in the main paper shows, among others, triad transition probabilities T assuming the triad exists in two consecutive terms. Here, apart from considering balanced and unbalanced triads, Fig. S6 includes a possibility that a triad will vanish in the next term. Transition probabilities among three states (balance, unbalanced, absence) were denoted as T_3. Triad disappearance is the main factor of triad changes for all tolerance values. For most Θ values, unbalanced triads are more likely to dissolve.

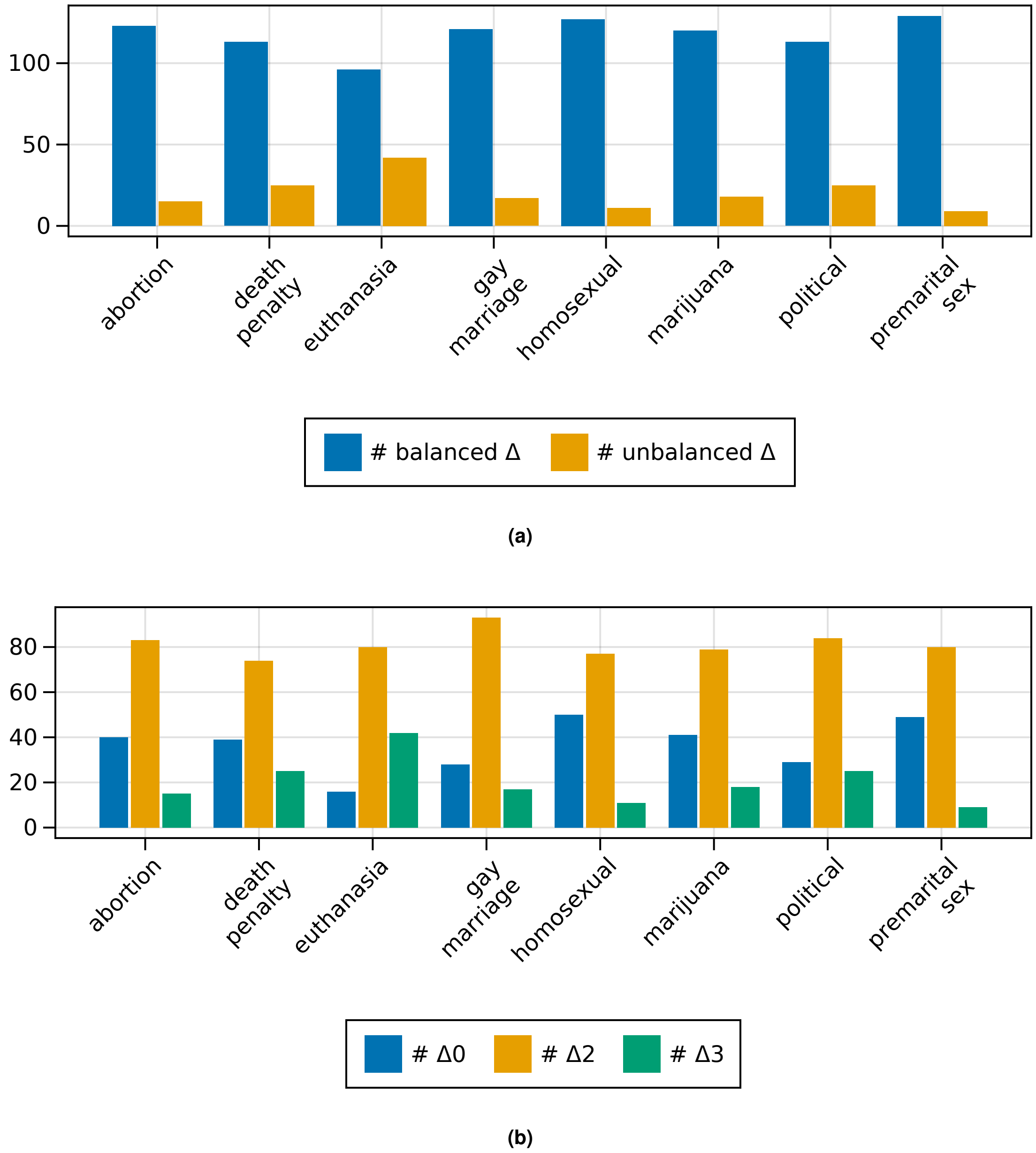

Figure S4. **Triad histograms for networks with uni-dimensional edges.** Panels show (a) exact numbers of balanced and unbalanced triads and (b) exact numbers of specific triad types in networks with signs obtained using a single topic.

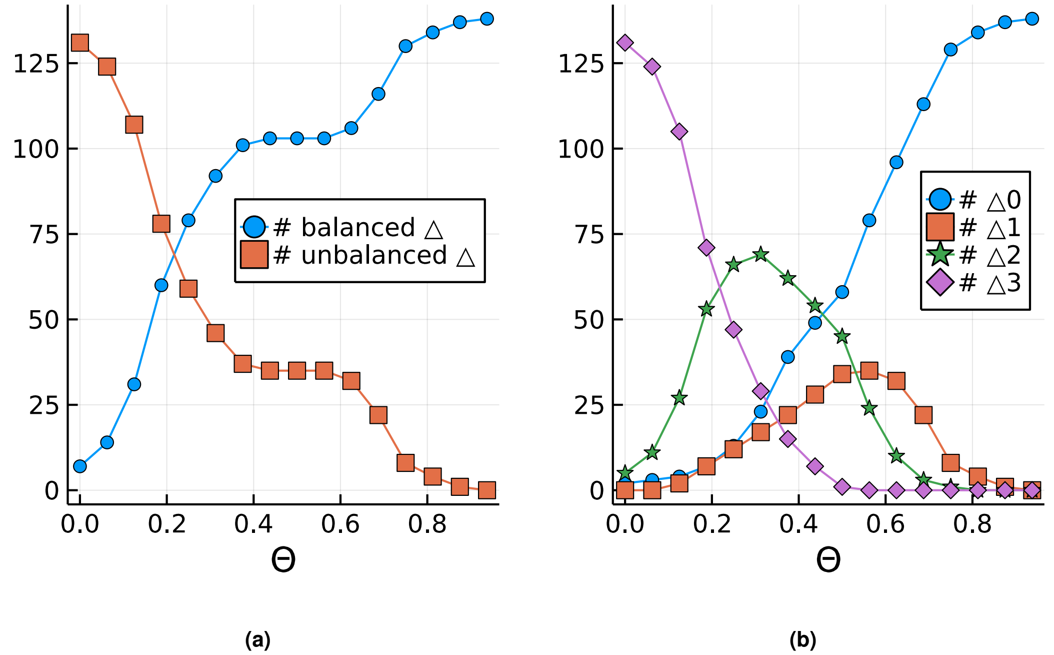

Figure S5. Triad histograms for networks with multidimensional attributes. Panels show (a) exact numbers of balanced and unbalanced triads and (b) exact numbers of specific triad types as a function of tolerance Θ taking all terms together.

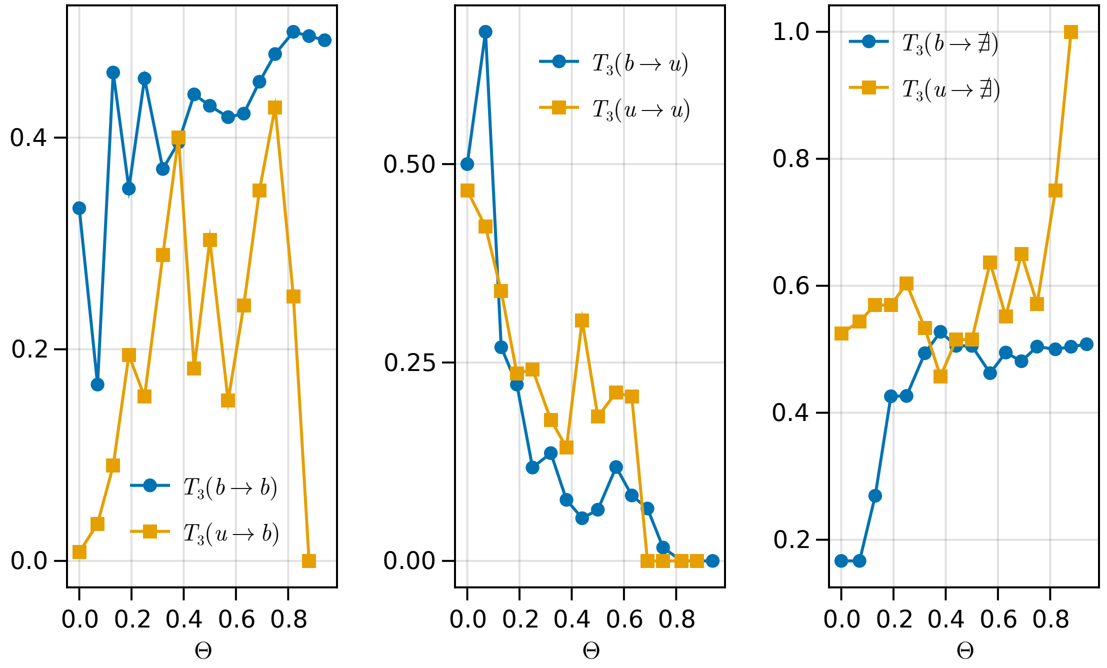

Figure S6. Transition probabilities of balanced and unbalanced triads including the possibility of triad's disappearance as a function of tolerance Θ. The left panel shows transitions to balanced triads, the center panel—to unbalanced triads, and the right panel shows the probability that the triad will vanish.

S4 Statistical testing of comparisons between data and random models

As mentioned in the main text, for models A_8 and C_8, the consecutive results in terms of Θ of densities of triads are correlated. The reason is as follows. In those models, students' attributes are shuffled either without (A_8) or with (C_8) keeping the vectors of attributes intact. Then, for each shuffling, the density of balanced triads as a function of tolerance is obtained. Therefore, the density for the specific Θ, $p_M(\Theta)$, will be similar to $p_M(\Theta \pm \Delta\Theta)$. Hence, the tests for specific Θ's are dependent on each other, prohibiting the use of family-wise errors.

Therefore, the goal was to create an *aggregated* test. Our reasoning is as follows. After each model realization, we obtain a curve $p_M(\Theta)$. We know the values of those curves only for the considered values of Θ (16 values in total). Hence, we need to compare the obtained values for each Θ separately. Thus, for each considered Θ, we ranked the obtained densities $p_M(\Theta)$. Then, the statistics for the randomized data set become the sum of ranks for different tolerances.

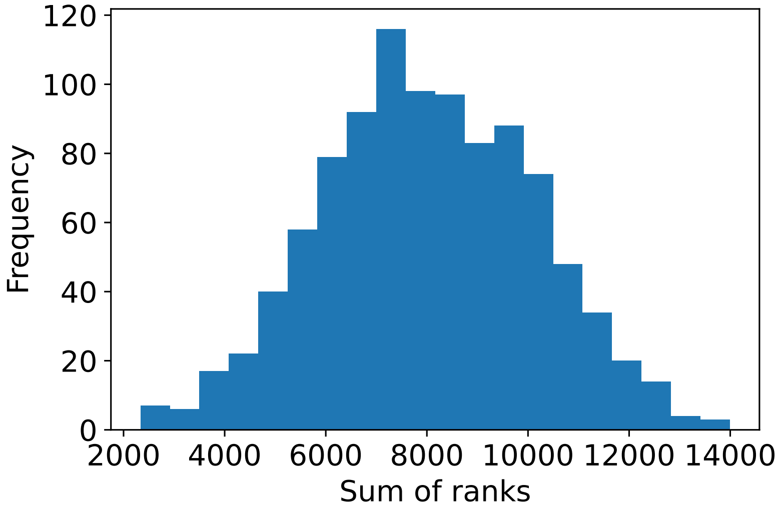

Figure S7. Histogram of values of statistics for the model C_8 in the first-level test. 1000 model realizations were used to obtain this figure. The value of the statistic for the real data curve was 13994.5.

Fig. S7 shows the histogram of the statistics for the model C_8. It is a bell-shaped distribution, and the Shapiro-Wilk test could not reject the hypothesis of the data normality (a p-value of 0.10 was obtained). However, as this type of test is non-parametric, we used the corresponding way to calculate the p-value in the test whether the model can describe the real data. We did not use methods assuming normality.

Thus, p-values of the tests whether the random model describes the data set well are defined by the probability that the random model yields a higher or equal sum of ranks for the plot of real data. For A_8 and C_8, we obtained p-values of 0.038 and 0.005, which allowed us to reject the corresponding null hypotheses.

A different case than presented above is for the model E_8. Here, for each tolerance, the density of positive links is obtained and this density is further used to generate new signs. As a result, for each tolerance, all the created networks are not directly related to each other and the family-wise error rate can be controlled.

S5 Static properties of triads for multi-edges without correlations

Having defined multi-edges with correlations removed (see section S2), we have performed similar comparisons to random models (A_8, E_8 and C_8) as presented in Fig. 4 in the main text. The results are presented in Fig. S8. The form of plots is different than in the main text because for the transformed variables, the number of considered tolerance values is much larger.

The comparison between the data and models A_8 and C_8 tells us that the curve for the real data is significantly larger with p-values of < 0.001 and 0.011, respectively. The specific identified ranges of tolerance values, which the model does not explain, are as follows:

- A_8: $\Theta \in [0.00, 0.30] \cup \{0.32, 0.33, 0.35, 0.40, 0.41, 0.68, 0.69, 0.71\} \cup [0.73, 0.78]$
- E_8: $\Theta \in [0.00, 0.14] \cup \{0.20\} \cup [0.25, 0.59]$
- C_8: $\Theta \in [0.00, 0.13] \cup \{0.20\} \cup [0.25, 0.30] \cup \{0.33, 0.35, 0.40, 0.41, 0.52, 0.57\}$

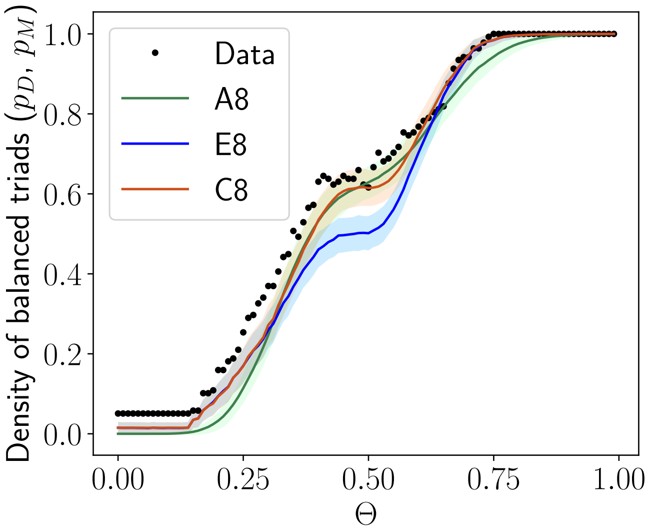

Figure S8. Comparison between the density of balanced triads in the case of multi-edges with correlations removed and random models A_8, E_8 and C_8. The shaded areas show one standard deviation.

Therefore, when using multi-edges without correlations, we obtain larger tolerance ranges of the cases of the random models being unable to explain the observed densities of balanced triads. However, the main difference between the obtained results for simple Manhattan-defined edges and multi-edges without correlations is the meaning of tolerance Θ. The results for both definitions of signed links are similar; thus, we preferred to show the results of the former in the main text because of the simplicity and the more natural tolerance interpretation.

S6 Comparing data and models with a small number of opinions

We tested models A, E and C in the case of varying numbers of opinions. The procedure steps were as follows:

1. Choose the number of attributes n.
2. From all attributes, choose a combination of n attributes.
3. Calculate true densities of balanced triads $p_D(\Theta;n)$.
4. Generate M random networks for models A_n, E_n, C_n and for each realization calculate densities of balanced triads $p_M(\Theta;n)$. M was chosen to be equal to 100.
5. Repeat steps (3-4) for all combinations of n attributes.
6. Repeat steps (2-5) for all possible numbers of attributes n.

In the rest of this section, we present the following results. First, we show the results of $p_D(\Theta;n)$ and $p_M(\Theta;n)$ for model A_n (Fig. S9). Similar plots for other models are analogous; therefore, they are not shown. Observations of curves $p_D(\Theta;n)$ and $p_M(\Theta;n)$ do not allow comparison of real and random networks. Similarly, as in the main paper, the difference $(p_D - p_M)$ is plotted (Fig. S10). To make the plots readable, we omitted error bars. As a consequence, one cannot assess if the differences are statistically significant. First-level tests for models A_n and C_n are presented in the main paper. Here, we present the 2nd level tests for models A_n and C_n and the tests for model E_n (Fig. S11-S13).

Fig. S9 shows that the tendencies of changes of densities of balanced triads are similar for the real and randomized networks. For small tolerance values, considering more and more topics, the density of balance triads becomes smaller. The opposite tendency is observed when $\Theta > 0.6$. These phenomena are caused by the fact that with low (high) tolerance values and the increasing number of attributes, negative (positive) links become more likely. Consequently, with increasing n, there appear to be more unbalanced (balanced) triads with three negative (positive) links. Fig. S9 presents the results only for model A_n. Plots for models E_n and C_n, not shown here, are analogous.

Fig. S10 shows a comparison between the real and randomized data sets for models A_n, E_n and C_n. For model A_n, one can observe a growth of the peak at $\Theta \approx 0.2$ with the increasing number of attributes. At the same time, the difference $(p_D - p_M)$

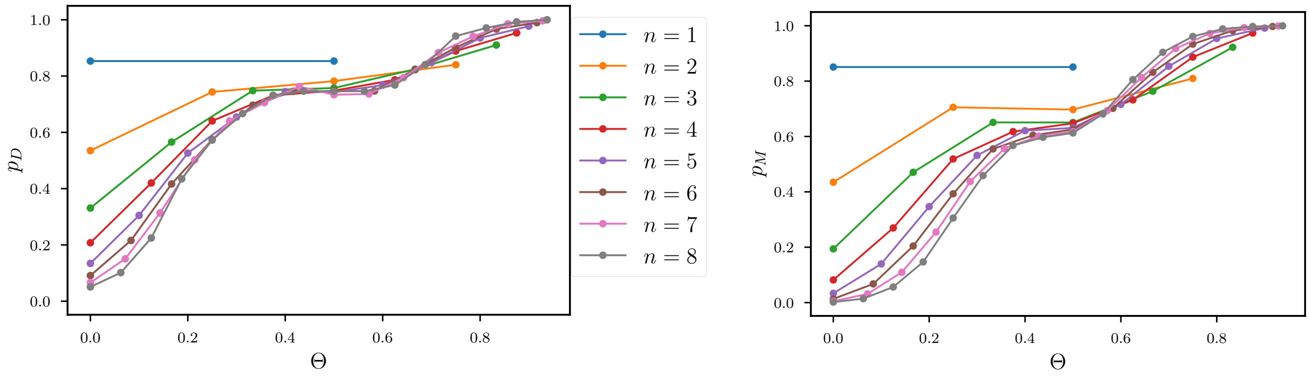

Figure S9. **Model A_n. Densities of balanced triads with varying numbers of attributes considered.** Panels show the densities for (a) real data set, $p_D(\Theta;n)$, and (b) for randomized ones, $p_D(\Theta;n)$. The legend for both panels is given in the middle. The lines are a guide for the eye. Error bars are not shown to increase plots' readability.

becomes negative (though not significantly) at $\Theta \approx 0.7$. Increasing values of the difference for small tolerances indicate that multidimensionality is essential to conclude that the model does not describe the real network well.

For model E_n, with the increase of n, the differences converge to the same shape. This shows that multidimensionality is not important to notice whether the model describes well the real network. Fig. S10b does not present results for E_1 because the model E_1 used in the main paper is slightly different from other E_n models. With a single attribute, only one type of unbalanced triads can be observed. In the main paper, model E_1 is constructed in such a way that this is preserved. When $n > 1$, all types of triads are possible. Therefore, here we present the results for multidimensional attributes only.

Fig. S10c presents the results of differences for model C_n. With increasing n, two peaks appear: at $\Theta \approx 0.2$ and at $\Theta \approx 0.75$. Again, multidimensionality is essential to differentiate the real network from the randomized ones.

Fig. S11 and S13 show the results of the second-level statistical analysis for specific tolerance values for models A_n and C_n. For these datasets, one-dimensional attributes do not allow the measurement of Heider balance interactions. Having two opinions, there are such attribute combinations that make this measurement possible. With more attributes, it becomes more evident for which tolerances Heider balance interactions can be observed and for which the real network does not differ from random ones.

Fig. S12 shows p-values of the hypothesis whether the observed densities $p_D(\Theta;n)$ are similar to $p_M(\Theta;n)$. Similarly, as for Fig. S10b, we conclude that multidimensionality does not play an important role for model E_n.

S7 Analyzing dynamic properties of data

The following subsections describe the details of the proposed agent-based model, the proposed error functions used in the fitting ABM procedure and to compare results between different models. The code is available here: https://github.com/pjgorski/NetHeider.

S7.1 Agent-based model details

The steps of the proposed agent-based model were described in the main paper. We examined a few model variants, including rewiring of edges (instead of removing and adding new ones) and changing the order of decisions. We presented the model yielding the best agreement with the data. Using the real data, we obtained the average probability of changing an unbalanced triad with one negative edge into a triad with three positive links (*i.e.*, p_n). Other parameters were difficult to calibrate because of many links vanishing in the real data, which could be caused by other factors (as stated in the main paper) unrelated to the analyzed model and dynamics. Therefore, the probability of choosing to remove a link p_r and the probability of adding a new connection p_{add} were fitted to best match the real data measures. The following values were evaluated: $p_r \in \{0, 0.05, 0.1, \dots, 0.3, 0.4, \dots, 1\}$ and $p_{add} \in \{0.01, 0.03, \dots, 0.09\}$. Another fitted parameter is the number of steps the ABM dynamics should run because it is unclear what this value should be. Therefore, during each simulation, we saved the network state every 5 steps up to 400 steps and later used these timestamps in the fitting procedure.

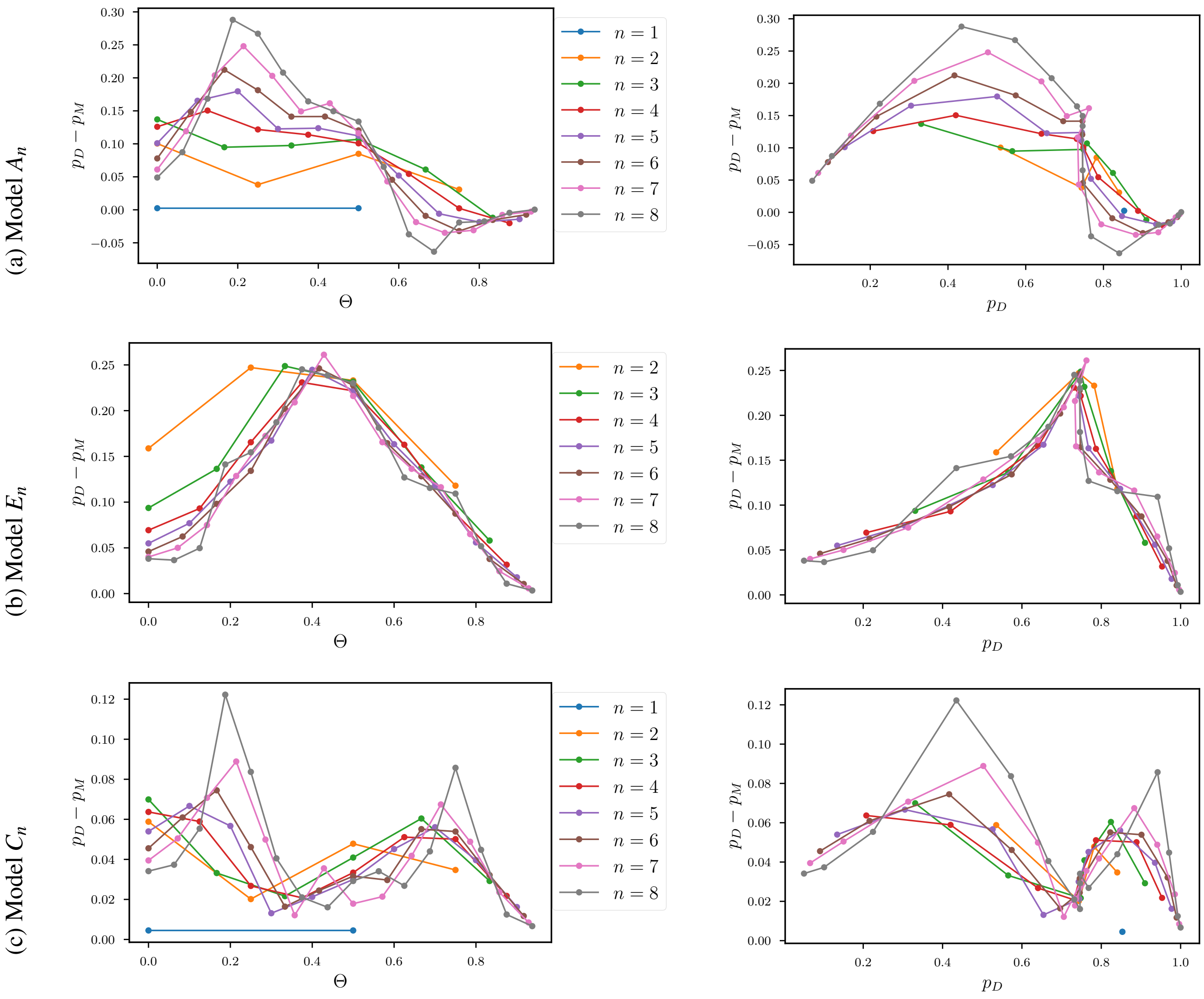

Figure S10. Density difference with varying numbers of attributes considered. Panels show the difference ($p_D - p_M$) between densities of balanced triads as a function of (left column) tolerance Θ or (right column) the density for the data set p_D. The legends for the panels are given in the middle. The lines are a guide for the eye. Error bars are not shown to increase plots' readability.

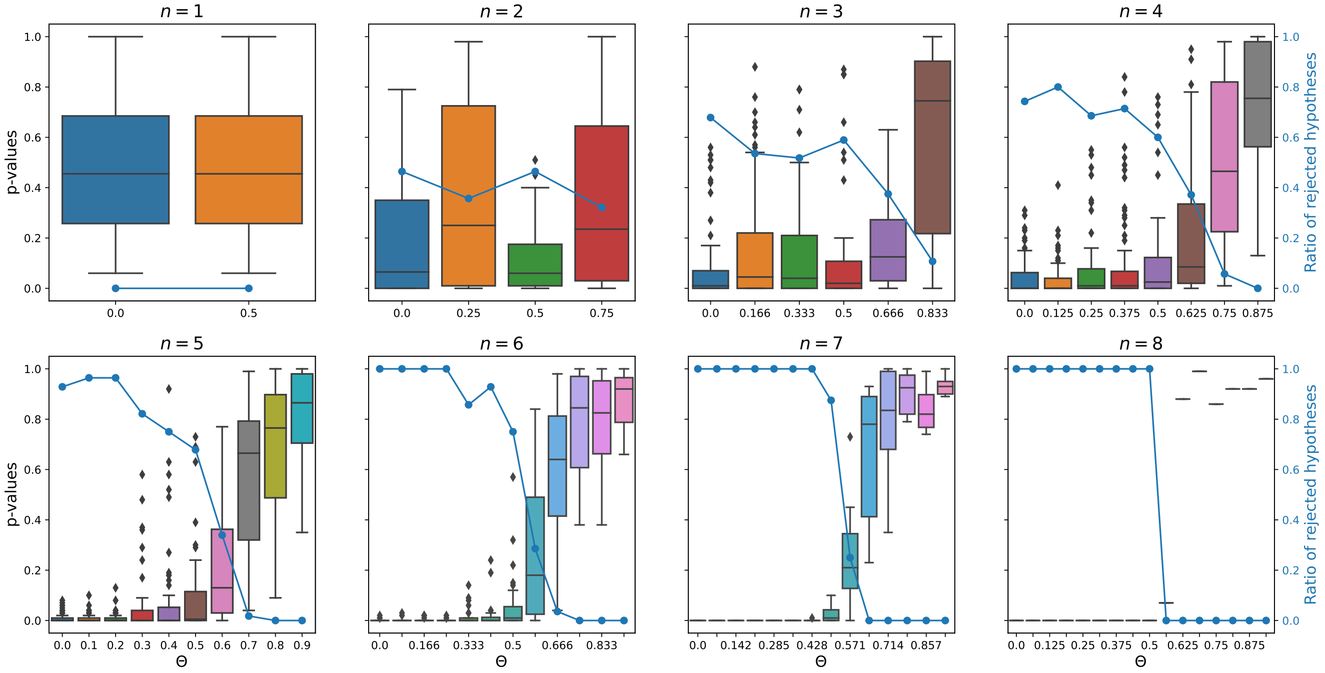

Figure S11. **Model A_n. Second-level statistical analysis with varying numbers of attributes considered.** Panels show the boxplots of p-values for specific tolerances Θ and the ratio of p-values leading to rejecting null hypotheses. Each panel corresponds to the specific number of attributes considered n.

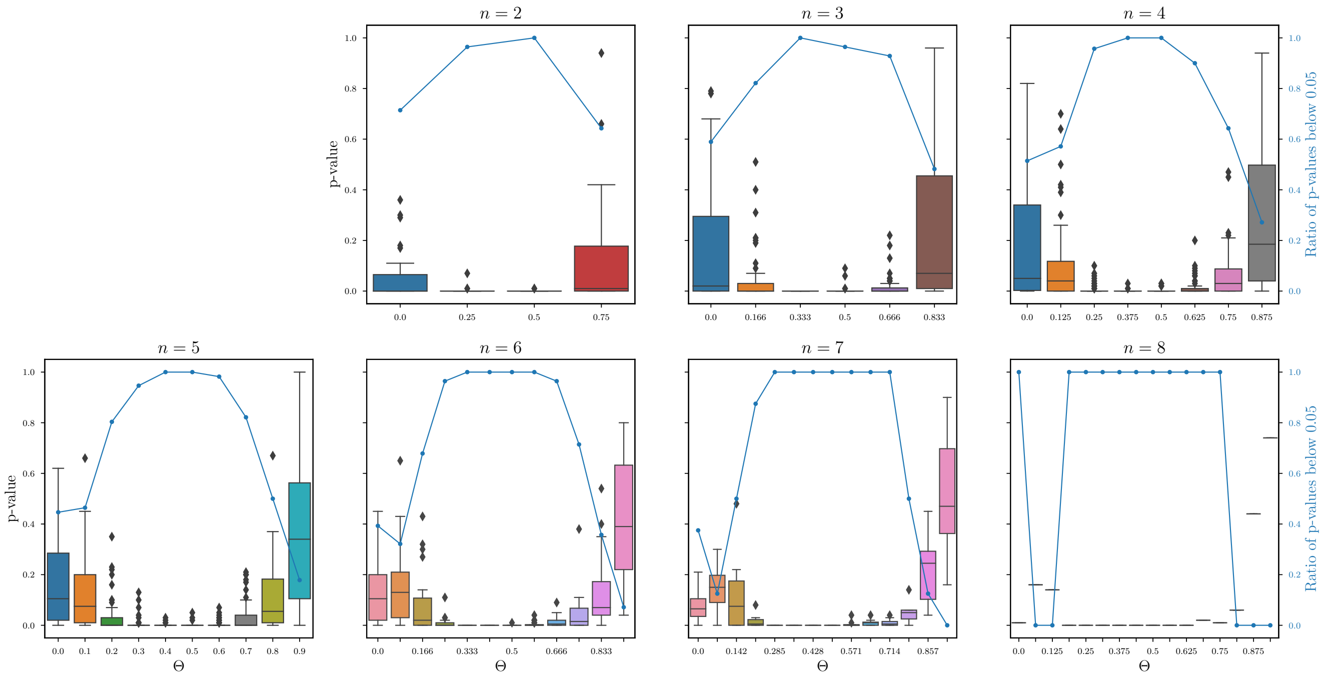

Figure S12. **Model E_n. Statistical analysis with varying numbers of attributes considered.** The panel for $n=1$ is not shown on purpose because the applied model E_1 differs from other models. See text for details.

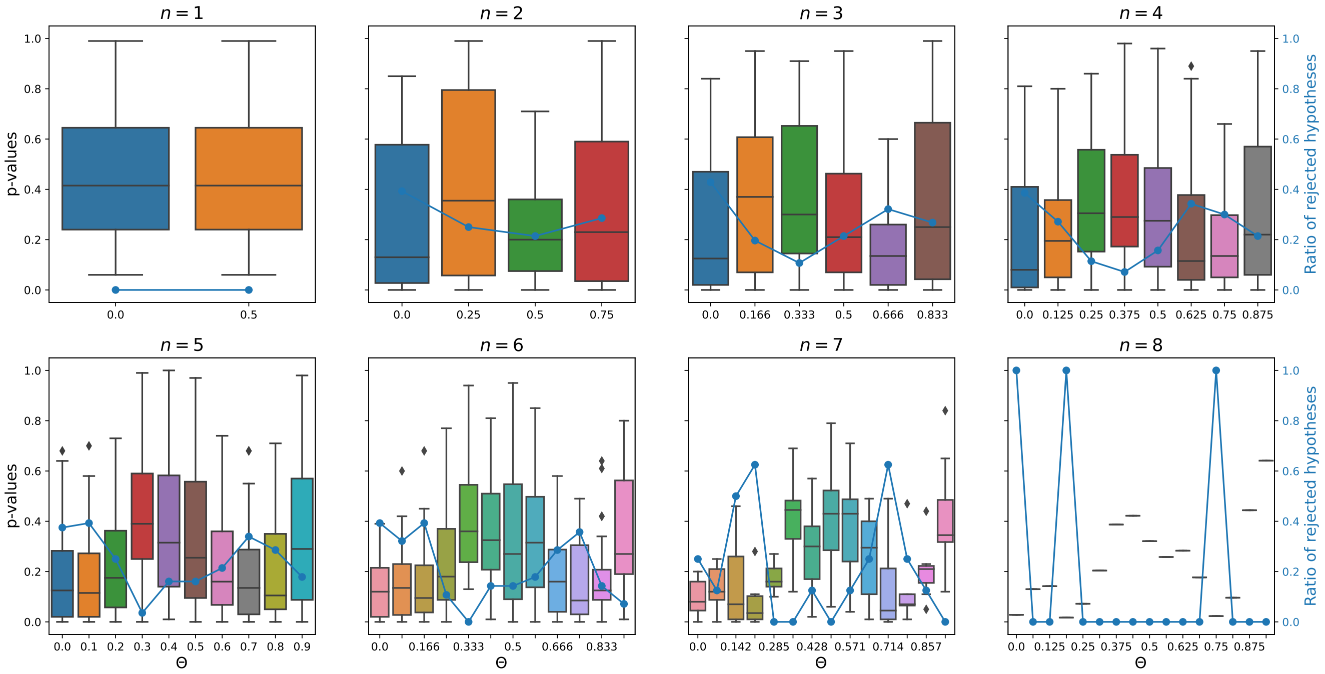

Figure S13. Model C_n. Second-level statistical analysis with varying numbers of attributes considered.

S7.2 Comparing transition probabilities obtained in real data to those obtained in models. Fitting procedure of ABM.

Fig. S3 shows the network graphs for three different tolerance values. Having very low or very high Θ results in very low numbers of balanced or unbalanced triads, respectively. For instance, for $\Theta = 0$, there are two positive edges, which form two balanced triads. These triads do not belong to the largest components. Only one of those triads is attached to an unbalanced triad, so only one triad may change. Therefore, the probability of transition from balanced to balanced triads $T(b \to b)$ may be in the range $[0.5, 1]$ and it explains the result obtained in Fig. 5b in the main paper. The proposed model is meaningful for metrics $T(b \to b)$ and $T(u \to b)$ when there are enough proper triads (balanced or unbalanced, respectively). Therefore, we propose the following error functions that omit such tolerance values for which the number of proper triads is too small to use our ABM.

$$E = \sum_{\Theta > 0.125} f\big(T_{b\to b}(\Theta), \hat{T}_{b\to b}(\Theta)\big) + \sum_{\Theta < 0.75} f\big(T_{u\to b}(\Theta), \hat{T}_{u\to b}(\Theta)\big), \tag{S2}$$

where T and $\hat{T}$ correspond to transition values from real data and from the model, respectively. As f, we used the mean squared error function.

We calculated an error function for each model parameter and for each considered timestamp. We obtained the smallest error value for the following set of parameters: $p_r = 0.15$, $p_{add} = 0.07$ with the number of steps $S = 75$. Obtained values of the error function are listed in Tab. S4.

**Model**	$\overline{E}$	$\delta\overline{E}$
**ABM with triadic interactions**	0.235	0.013
**Node-randomized**	0.625	0.015
**Edge-randomized**	0.732	0.017
**ABM with dyadic interactions**	0.290	0.011

Table S4. Comparison of mean error value $\overline{E}$ and its uncertainty $\delta\overline{E}$ between different models. "ABM with triadic interactions" is the ABM introduced in this paper. "Node-randomized" and "edge-randomized" are two proposed randomizations. "ABM with dyadic interactions" is the ABM introduced in[2].

Also, using the eq. (S2), we calculated the difference between the transitions in real data and those obtained in the two proposed randomizations. The obtained error values are significantly larger than the value yielded by our ABM (see Tab. S4).

S7.3 Agent-based model with only dyadic interactions

Ref.[2] showed that dyadic interactions may be enough to explain observed triadic statistics. Therefore, we evaluated the proposed ABM with dyadic interactions and compared the measured transitions. The model contains two parameters: α and β. α defines the type of dyadic interactions. Having $\alpha = 1$, only positive dyadic interactions matter; having $\alpha = 0$, only negative dyadic interactions matter; and having $\alpha = 0.5$, both positive and negative relations influence the changes to the same extent. Parameter β is the dynamics parameter (inverse temperature) that defines randomness in the dynamics. Large and small β correspond to large or little randomness in the dynamics.

Using our MSE error function, we simulated the model with different parameter values. We evaluated the following ranges: $\alpha \in \{0.5, 1\}$ and $\beta \in \{0, 0.25, \ldots, 5\}$. Similarly as previously, we did not define the number of steps but checked results at different values of this parameter. The smallest error value was obtained for the following parameters: $\alpha = 1$, $\beta = 5$ and $S = 230$ ABM steps. As seen in Tab. S4, this is significantly larger than for our ABM with triadic interactions.

References

1. Nigam, A. *et al.* ONE-M: Modeling the Co-evolution of Opinions and Network Connections. In *Proc. ECML-PKDD*, 122–140, DOI: https://doi.org/10.1007/978-3-030-10928-8_8 (Springer, Dublin, Berlin, 2018).

2. Pham, T. M., Korbel, J., Hanel, R. & Thurner, S. Empirical social triad statistics can be explained with dyadic homophylic interactions. *Proc. Natl. Acad. Sci.* **119**, e2121103119, DOI: https://doi.org/10.1073/pnas.2121103119 (2022).
